# A Randomized Pilot Study of L-Arginine Infusion in Severe Falciparum Malaria: Preliminary Safety, Efficacy and Pharmacokinetics

**DOI:** 10.1371/journal.pone.0069587

**Published:** 2013-07-29

**Authors:** Tsin W. Yeo, Daniel A. Lampah, Indri Rooslamiati, Retno Gitawati, Emiliana Tjitra, Enny Kenangalem, Ric N. Price, Stephen B. Duffull, Nicholas M. Anstey

**Affiliations:** 1 Global Health Division, Menzies School of Health Research and Charles Darwin University, Darwin, NT, Australia; 2 Menzies School of Health Research-National Institute of Health Research and Development Research Program, and District Ministry of Health, Timika, Papua, Indonesia; 3 National Institute of Health Research and Development, Jakarta, Indonesia; 4 Division of Medicine, Royal Darwin Hospital, Darwin, NT, Australia; 5 Centre for Tropical Medicine, Nuffield Department of Clinical Medicine, University of Oxford, Oxford, United Kingdom; 6 School of Pharmacy, University of Otago, Dunedin, New Zealand; Naval Medical Research Center, United States of America

## Abstract

**Background:**

Decreased nitric oxide (NO) and hypoargininemia are associated with severe falciparum malaria and may contribute to severe disease. Intravenous L-arginine increases endothelial NO in moderately-severe malaria (MSM) without adverse effects. The safety, efficacy and pharmacokinetics of L-arginine or other agents to improve NO bioavailability in severe malaria have not been assessed.

**Methods:**

In an open-label pilot study of L-arginine in adults with severe malaria (ARGISM-1 Study), patients were randomized to 12 g L-arginine hydrochloride or saline over 8 hours together with intravenous artesunate. Vital signs, selected biochemical measures (including blood lactate and L-arginine) and endothelial NO bioavailability (using reactive hyperemia peripheral arterial tonometry [RH-PAT]) were assessed serially. Pharmacokinetic analyses of L-arginine concentrations were performed using NONMEM.

**Results:**

Six patients received L-arginine and two saline infusions. There were no deaths in either group. There were no changes in mean systolic (SBP) and diastolic blood pressure (DBP) or other vital signs with L-arginine, although a transient but clinically unimportant mean maximal decrease in SBP of 14 mmHg was noted. No significant changes in mean potassium, glucose, bicarbonate, or pH were seen, with transient mean maximal increases in plasma potassium of 0.3 mmol/L, and mean maximal decreases in blood glucose of 0.8 mmol/L and bicarbonate of 2.3 mEq/L following L-arginine administration. There was no effect on lactate clearance or RH-PAT index. Pharmacokinetic modelling (n = 4) showed L-arginine concentrations 40% lower than predicted from models developed in MSM.

**Conclusion:**

In the first clinical trial of an adjunctive treatment aimed at increasing NO bioavailability in severe malaria, L-arginine infused at 12 g over 8 hours was safe, but did not improve lactate clearance or endothelial NO bioavailability. Future studies may require increased doses of L-arginine.

**Trial Registration:**

ClinicalTrials.gov NTC00616304

## Introduction

The outcome of adults with severe malaria has improved with use of intravenous artesunate compared to quinine [1], [2]. However, case-fatality rates remain as high as 30%, with no survival benefits noted in the first 48 hours of therapy in adults [1]. Adjunctive agents targeting underlying pathogenesis may be needed to further improve the outcome. Key mechanisms underlying the pathogenesis of severe and fatal falciparum malaria are microvascular obstruction and impaired organ perfusion resulting from cytoadherence of parasitized red cells to activated endothelial cells, impaired microvascular reactivity and endothelial dysfunction associated with impaired bioavailability of endothelial nitric oxide [3], [4], [5], [6].

Nitric oxide (NO) bioavailability is reduced in both African children with cerebral malaria [7], and Asian and Melanesian adults with severe falciparum malaria [8]. Mechanisms which may decrease endothelial NO bioavailability in severe malaria include impaired nitric oxide synthase 2 (NOS2) expression [7], hypoargininemia [8], [9], [10], quenching by cell-free haemoglobin [11], and increased concentrations of the nitric oxide synthase inhibitor, asymmetrical dimethylarginine (ADMA) [12]. Endothelial NO production relies on movement of L-arginine from the extracellular to intracellular compartment using the cationic amino acid transporter-1 (CAT-1) with a half-saturating concentration (*Km*) of 100–150 µmol/L [13]. Plasma L-arginine concentrations in severe malaria are low in both African children and Asian adults and increase with clinical recovery [8], [9], [10]. We have previously shown that infusion of L-arginine in doses of 3 g, 6 g and 12 g over 30 minutes in adults with moderately severe malaria (MSM) increased endothelial and pulmonary NO bioavailability in a dose-dependent manner with minimal adverse effects [8], [14]. Pharmacokinetic modelling of L-arginine in MSM demonstrated the half-life of exogenous L-arginine was significantly reduced compared to healthy adults [15]. Simulations of dosing schedules predicted a L-arginine dose of 12 g given over 8 hours would result in concentrations above the *Km* of CAT-1 in 75% of patients [15].

There have been no previous clinical studies of agents to improve NO bioavailability in severe malaria. Based on the safety, efficacy and pharmacokinetic findings in moderately severe malaria, we conducted a study of intravenous L-arginine infusion in Indonesian adults with severe malaria (ARGISM-1 Study). We evaluated the safety and efficacy profile, including the changes on vital signs, electrolytes and acid base status, lactate clearance and endothelial NO bioavailability, and performed a preliminary pharmacokinetic analysis.

## Methods

### Study Site, Participants and Ethics Statement

The protocol for this trial and supporting CONSORT checklist are available as supporting information; see Checklist S1 and Protocol S1. The ARGISM-1 study was conducted at Mitra Masyarakat Hospital, Timika, Papua, Indonesia, an area with unstable malaria transmission. Written informed consent was obtained from patients or relatives if they were comatose or too ill. The study was approved by the ethics committees of the National Institute of Health Research and Development, Indonesia, and the Menzies School of Health Research, Australia. The study was registered at clinicaltrials.gov as NTC00616304. The funders had no role in the study design, data collection, analysis, decision to publish or preparation of the manuscript.

Patients from 18–60 years of age with severe malaria (SM) were enrolled. SM was defined as having ≥1 modified WHO severity criteria including i) acute renal failure (creatinine>265 µmol/L), ii) hyperbilirubinemia (total bilirubin>50 µmol/L with either renal impairment (creatinine>130 µmol/L), or parasitemia of ≥100,000/µL), iii) blackwater fever, iv) hyperparasitemia (>10% parasitized red cells), v) cerebral malaria (Glasgow Coma Score<11), vi) hypoglycemia (glucose<2.5 mmol/L), vi) respiratory distress (respiratory rate>32/min, and the presence of *Plasmodium falciparum* parasitemia as used previously [8], [16]. Clinical criteria for exclusion included pregnancy or breastfeeding, systolic blood pressure of <90 mmHg after fluid resuscitation, having received anti-malarials longer than 18 hours prior to admission, significant comorbidities (including diabetes mellitus, and pre-existing cardiac, renal or hepatic disease), known allergies to L-arginine, clinical evidence of bacterial infection, current use of spironolactone, oral nitrates, phosphodiesterase inhibitors, alpha-blocking antihypertensive agents, and L-arginine. Hematological and biochemical exclusions were hemoglobin levels of <60 g/L, venous potassium >5.5 meq/L, chloride >110 meq/L, and bicarbonate <15 meq/L.

### Study Design

All patients with suspected severe malaria were identified at the emergency department or outpatient clinics. On confirmation of severe malaria, patients were screened for any exclusion criteria, and if negative, informed consent was obtained. Patients were then randomized in a 2∶1 ratio to receive either L-arginine or placebo with allocation concealed in a sealed opaque envelope. The randomization sequence and envelopes were prepared by individuals not directly involved in the study. After randomization, patients underwent a standardized history and physical examination and were monitored in a high dependency unit for at least 24 hours after the start of intravenous L-arginine or saline infusion. L-arginine hydrochloride (Phebra, Sydney, Australia) was administered as a 12 g dose diluted to 10% in normal saline and given by an infusion pump over 8 hours, with a similar volume of normal saline being used in patients randomized to placebo. Patients had continuous electrocardiogram monitoring throughout and up to one hour after the end of the infusion. Vital signs including systolic (SBP) and diastolic blood pressure (DBP), respiratory rate (RR), pulse rate (PR), Glasgow Coma Score (GCS) and temperature were recorded at regular intervals during and after infusions. Oxygen saturation and blood methemoglobin concentrations were measured using a pulse co-oximeter (Masimo Rad 7, California). Hemoglobin and leukocyte counts were measured by Coulter counter at baseline and venous blood was drawn into lithium heparin tubes at 0, 1, 2, 4, 8, 9, 12 and 24 hours after the start of infusion. A hand held biochemical analyzer (i-STAT Corp) was used to measure venous potassium, chloride, bicarbonate and lactate at these time points [14]. Endothelial function was measured non-invasively at 0, 1, 8, 9 and 24 hours by the change in pulse wave amplitude of the digits by peripheral arterial tonometry (EndoPAT) in response to reactive hyperemia, giving a RH-PAT Index as described previously [8]. The pulse wave amplitude in RH-PAT is at least 50% dependent on endothelial NO production and internal validation and repeatability of RH-PAT in this population have previously been reported [8], [17]. RH-PAT is also associated with lactate in severe malaria suggesting it may also reflect function in the systemic microvasculature [8]. We also measured microvascular reactivity using near infrared resonance spectroscopy as previously described [6]. All patients were managed by non-study hospital physicians and treated with intravenous artesunate and antibiotics using standard hospital protocols and were followed up until discharge.

Pre-specified criteria for cessation of the infusions were extravasation, a decrease in the SBP of >25 mmHg from baseline or to below 80 mmHg, venous potassium of ≥6.5 meq/L or ECG changes consistent with hyperkalemia, anaphylaxis, a venous blood glucose <3 mmol/L, bicarbonate <10 meq/L, pH <7.1 or methemoglobin level >12%.

### L-arginine Concentration Measurements

Venous blood was centrifuged within 20 minutes to obtain plasma and immediately stored at −70°C. Amino acids were extracted from 50 µl of plasma after the addition of 50 µl of an internal standard (norleucine) and 200 µl of cold ethanol. Deproteinized plasma was derivatized with AccQFluor reagent (Waters Corp., Milford, MA), and amino acids were measured by high-performance liquid chromatography (Shimadzu, Kyoto, Japan) as previously described [8]. The limits of detection were 1.6 µM, and the limit of quantification was 4.9 µM. Inter and intraday coefficient of variation was 4.1 and 1.2.

### Population Pharmacokinetic Modelling

We used a model derived from a previous study of moderately severe malaria which was a two compartmental model with first order elimination [15], as an initial construct to explore the data from the patients with severe malaria. A first order conditional estimation method with interaction was used to analyse the data using NONMEM (version 6), with the G77 FORTRAN compiler. Model suitability was evaluated with standard goodness-of-fit criteria including objective function, parameter estimates, between-subject variability and diagnostic plots. Compartmental models were parameterized in terms of volumes of distribution (V), and clearances.

### Statistical Methods

Mean values of the vital signs and biochemical parameters before infusion were compared to mean values obtained during and after infusion using a paired t-test (for continuous variables with a normal distribution or variables log transformed to a normal distribution). If parameters were not normally distributed, values before and after infusion were compared by the Wilcoxon Signed Rank Test. Because analysis in this manner could mask transient but potentially clinically significant changes in a minority of subjects, we also analyzed the maximum change in the vital signs and biochemical markers at any time during and up to two hours after L-arginine infusion compared to baseline readings. This gave a “worst-case scenario”, however transient, for each marker. The mean of the pre-infusion values in each patient was subtracted from the lowest (SBP, DBP, glucose, bicarbonate, pH) and highest (pulse rate, respiratory rate, potassium, chloride and anion gap) measurement during infusion or in the two hours following infusion and expressed as mean maximum change. Relationships between maximum change and the dose weight ratio (calculated as mg/kg) were assessed by Spearman’s rank correlation coefficient (ρ). Sample size calculation estimated 20 patients should be randomized to L-arginine and 8 to saline to detect a 20 per cent difference in endothelial function. All analyses were performed with Stata 11 (Statacorp). A two sided value of p<0.05 was considered significant.

## Results

### Patients

**Figure 1:**
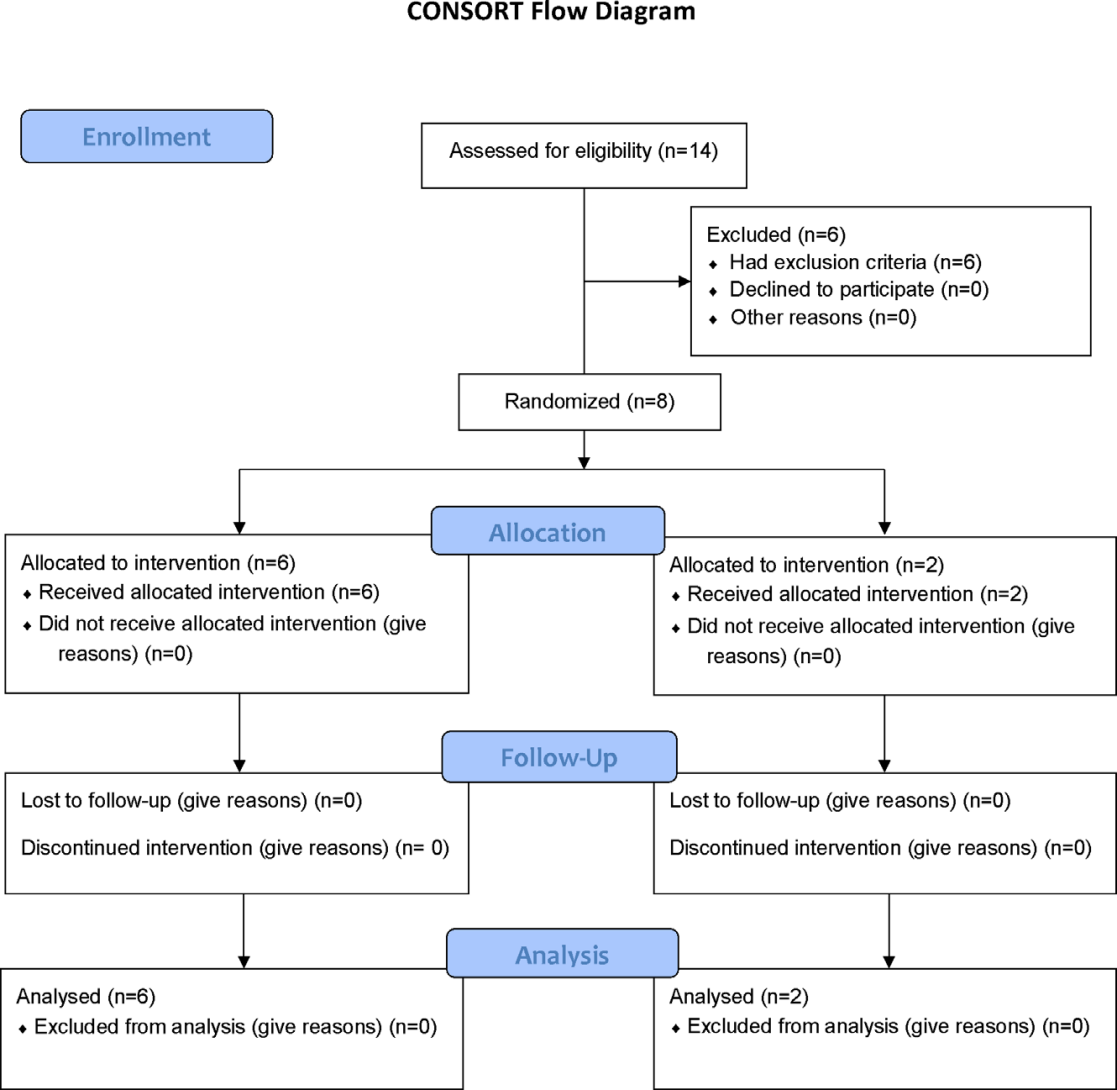
Flow diagram of patients screened and enrolled for the ARGISM-1 study.

Eight patients were enrolled in the study from 1^st^ February 2008 to 28^th^ February 2009; six received L-arginine and two, saline **(** **Figure 1** **)**. Local circumstances, including difficulties in accessing the field site, prevented continuation of the study but did not affect the preceding conduct of the study. In patients who received L-arginine, four had one severity criterion (three with cerebral malaria and one with hyperbilirubinemia), one had two criteria (acute renal failure and hyperbilirubinemia) and one had four severity criteria (cerebral malaria, acute renal failure, hyperbilirubinemia, and blackwater fever). In those who received saline, one patient had one severity criterion (cerebral malaria) and one had four criteria (cerebral malaria, respiratory distress, hyperbilirubinemia, and hyperparasitemia). All the patients were treated with intravenous artesunate and six received additional antibiotics. One patient with acute renal failure received peritoneal dialysis but went on to recover renal function, and there were no deaths in the study. The patients’ baseline details are shown in **Table 1** **.**

**Table 1:** Baseline characteristics and laboratory results in severe malaria patients.

	L-arginine Infusion Group	Saline Infusion Group
Number of patients	6	2
Mean age (year; range)	31.5 (18 to 55)	35.5 (27 to 44)
No (%) of males	4 (66%)	2 (100%)
Mean weight (kg; range)	59 (45 to 70)	57.5 (55 to 60)
No (%) of Papuan Highlanders	4 (66%)	2 (100%)
Mean no of days before admission (range)	1.2 (1 to 2)	1 (1 to 1)
Mean systolic blood pressure (mmHg; range)	115 (94 to 141)	106 (98 to 114)
Mean pulse rate (beats/minute; range)	87 (74 to 99)	88 (81 to 95)
Mean respiratory rate (breaths/min; range)	23 (21 to 25)	28 (24 to 32)
Mean temperature (°C; range)	36.9 (36.3 to 38)	36.3 (36.2 to 36.4)
Mean methemoglobin level (%; range)	1.2 (0.9 to 1.8)	1.2 (1.1 to 1.3)
Mean leukocyte count (10^3^/µL, range)	10.3 (7.4 to 11.9)	9.1 (7.1 to 11.1)
Mean haemoglobin count (g/dl, range)	13.2 (12.9 to 14.3)	13.7 (11.6 to 16.3)
Mean L-arginine plasma concentration (µmol/L, range)	56 (32 to 95)	62 (48 to 76)
Mean lactate concentration (mmol/L, range)	2.3 (1.2 to 3.3)	3.8 (3.3 to 4.4)
Mean bicarbonate concentration (mmol/L; range)	22.7 (17.4 to 26.9)	20.7 (18.6 to 22.8)
Mean potassium concentration (mmol/L; range)	3.8 (3.5 to 4.1)	3.8 (3.8 to 3.8)
Mean glucose concentration (mmol/L; range)	6.6 (4 to 9.2)	6.85 (5.6 to 8.1
Mean plasma phosphate (meq/L; range)	1.2 (0.6 to 2.5)	1.1 (0.7 to 1.5)
Geometric mean parasite density (parasites/µL; range)	42300 (6660 to 148296)	258505 (95359 to 700848)

### Clinical Adverse Effects during L-arginine Infusion

There were no significant clinical adverse effects in any patient; in particular, there were no allergic reactions, convulsions, pain, rash, swelling or erythema at the infusion site. In the two patients who were not comatose, there was no increase in severity of nausea, vomiting, headache or prostration during or after the L-arginine infusion.

### Effect of L-arginine on Vital Signs, Cardiac Rhythm, Concentrations of Methemoglobin

**Figure 2:**
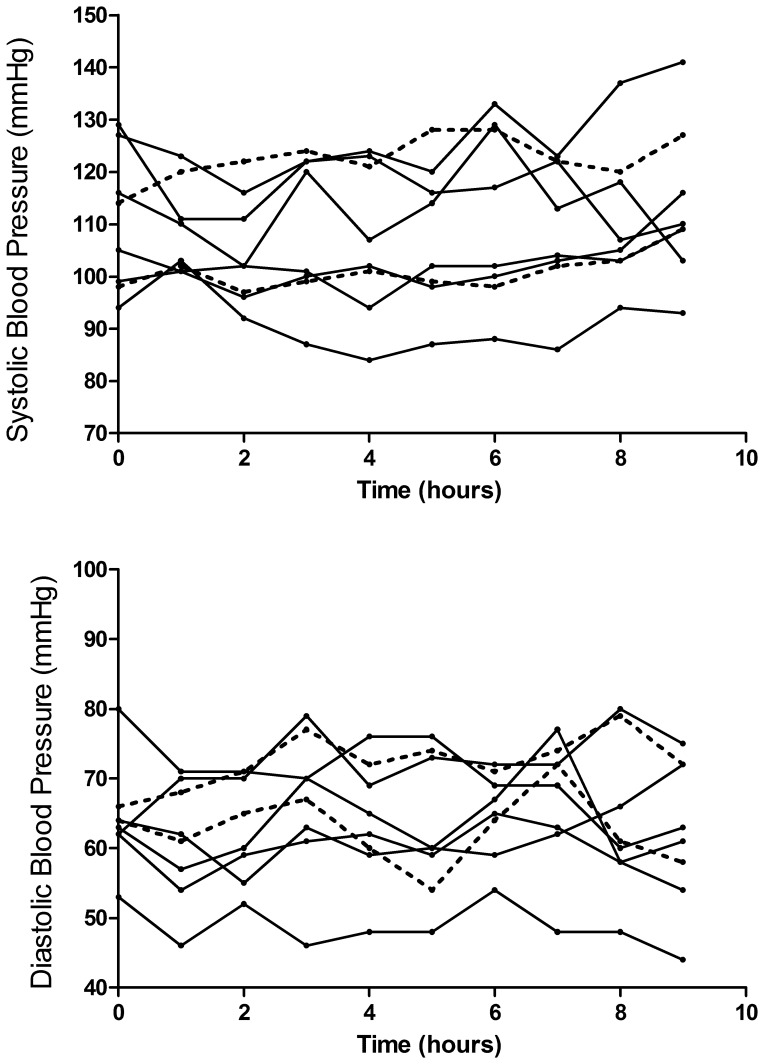
a. Profile of the systolic blood pressure of patients before, during and one hour after receiving L-arginine. Dotted lines indicate patients who received saline. L-arginine was infused between zero and eight hours. b. Profile of the diastolic blood pressure of patients before, during and one hour after receiving L-arginine. Dotted lines indicate patients who received saline.

There was no difference in coma recovery times between patients who received L-arginine (mean 35 hours [range 12–69 hours]) and those who received saline (34 and 40 hours). There was no significant effect on the mean pulse and respiratory rate before and after infusion of L-arginine and there were no notable changes on the electrocardiogram. There were no clinically significant changes in the mean systolic or diastolic blood pressure during and up to 16 hours after the end of the infusion in patients who received L-arginine (**Figure 2A and 2B** **)** or saline. However, the *mean maximum change* in the systolic blood pressure during the infusion in those who received L-arginine was-14 mmHg (range −11 to −19 mmHg) (paired T-test, p<0.001) compared to −0.5 mmHg (range 0 to −1 mmHg) with saline; **Table 2** **.** The change in the mean maximal diastolic blood pressure in those receiving L-arginine was −12 mmHg, (range −6 to −16 mmHg) (paired T-test; p = 0.003) compared to −6 mmHg (range −2 to −10 mmHg) with saline; **Table 2**. There was a significant correlation between the maximal fall in systolic (ρ = −0.82, p = 0.04) and diastolic (ρ = −0.85, p = 0.03) blood pressure with increasing mg per kg L-arginine doses. There were no differences noted in the maximum changes in pulse rate, respiratory rate and methemoglobin levels between the groups.

**Table 2:** Maximum change in parameters at any time during and up to one hour after L-arginine infusion.

	L-arginine	Saline
SBP (mmHg)	−14 (−11 to −20)	−1 (−1 to −1)
DBP (mmHg)	−12 (−6 to −15)	−6 (−2 to −10)
Potassium (mmol/L)	0.3 (0.2 to 0.7)	−0.1 (−0.2 to −0.1)
Bicarbonate (meq/L)	−2.3 (0 to −4.4)	0.5 (−1 to 2)
pH	−0.02 (−0.007 to −0.04)	0.03 (0 to 0.07)
Chloride (mmol/L)	2.8 (2 to 3)	2.5 (0 to5)
Glucose (mmol/L)	−0.8 (−1.4 to −0.1)	1.05 (0.4 to 1.7)

Mean maximum change (range).

### Effect of L-arginine on Electrolytes, pH and Glucose

**Figure 3:**
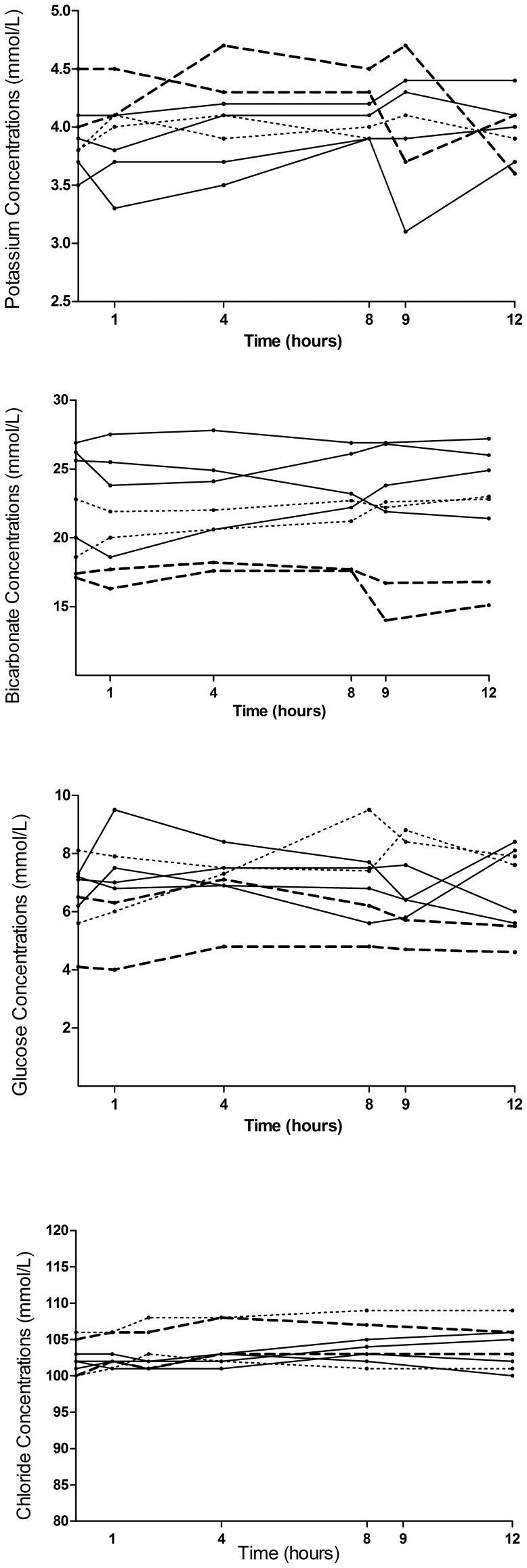
a. Profile of the venous potassium concentration of patients before, during and three hours after receiving L-arginine. Broken lines indicate patients with acute renal failure and dotted lines indicate patients who received saline. L-arginine was infused between zero and eight hours. b. Profile of the venous bicarbonate concentrations of patients before, during and three hours after receiving L-arginine. Broken lines indicate patients with acute renal failure and dotted lines indicate patients who received saline. c. Profile of the venous blood glucose concentrations of patients before, during and three hours after receiving L-arginine. Broken lines indicate patients with acute renal failure and dotted lines indicate patients who received saline. d. Profile of the venous blood chloride concentrations of patients before, during and three hours after receiving L-arginine. Broken lines indicate patients with acute renal failure and dotted lines indicate patients who received saline.

There were no clinically significant changes in the mean values of potassium, chloride, glucose, pH, anion gap and bicarbonate during and up to 16 hours after infusion compared to pre-infusion levels; **Figure 3A, 3B, 3C and 3D**. Significant but transient and clinically unimportant differences (by paired T-test) were noted in the maximal change of potassium, chloride and glucose following L-arginine infusion, **Table 2**. The mean baseline bicarbonate concentrations or pH in those that received L-arginine or saline are shown in **Table 1** and the maximal decreases in bicarbonate and pH in **Table 2**. There was no relationship noted between the maximal change in potassium, chloride, glucose or bicarbonate with increasing mg per kg L-arginine doses.

### Effect of L-arginine on Lactate Clearance and Endothelial Function

**Figure 4:**
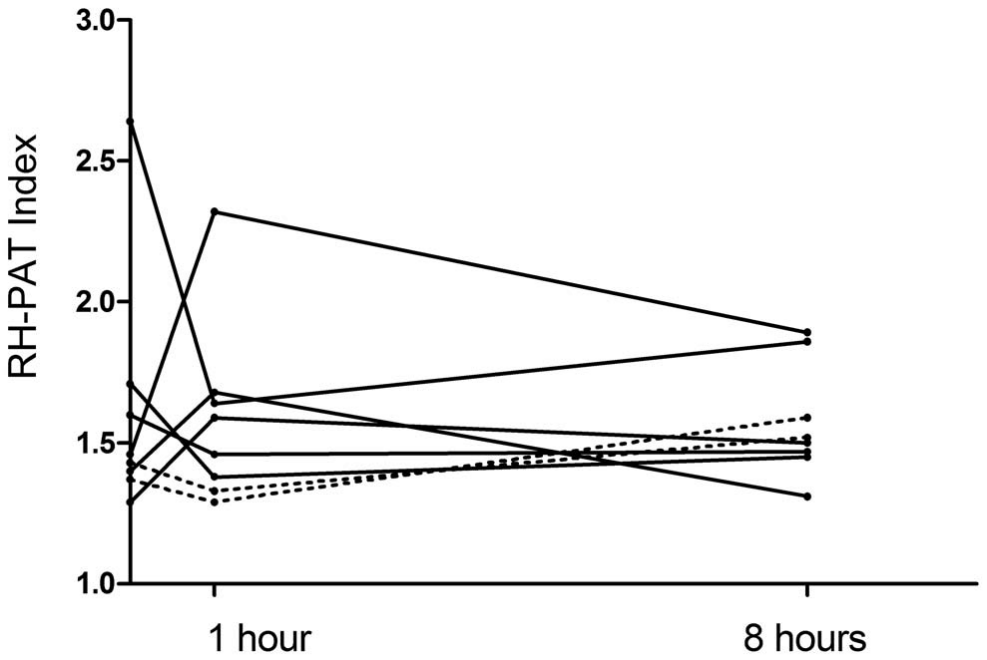
RH-PAT index profile pre-infusion, one hour and eight hours after infusion of L-arginine. Dotted lines indicate patients who received saline.

Only 5 patients (4 L-arginine and 1 saline) had elevated blood lactate (>2 mmol/L) at baseline, with the baseline lactate being higher in the two patients randomized to receive saline **Table 1**. The lactate clearance rate did not appear to be different between the patients who received L-arginine or saline, however the small numbers of patients did not allow us to formally compare the groups. Six patients (4 L-arginine and 2 saline) had impaired endothelial function (defined as RH-PAT index <1.67) at baseline. There was also no difference in the change in endothelial function during and up to one hour after infusion of L-arginine (p = 0.3) or saline compared to baseline; **Figure 4**. There was also no change in microvascular reactivity during and after L-arginine infusion.

### Preliminary Pharmacokinetics of L-arginine after Infusion

**Figure 5:**
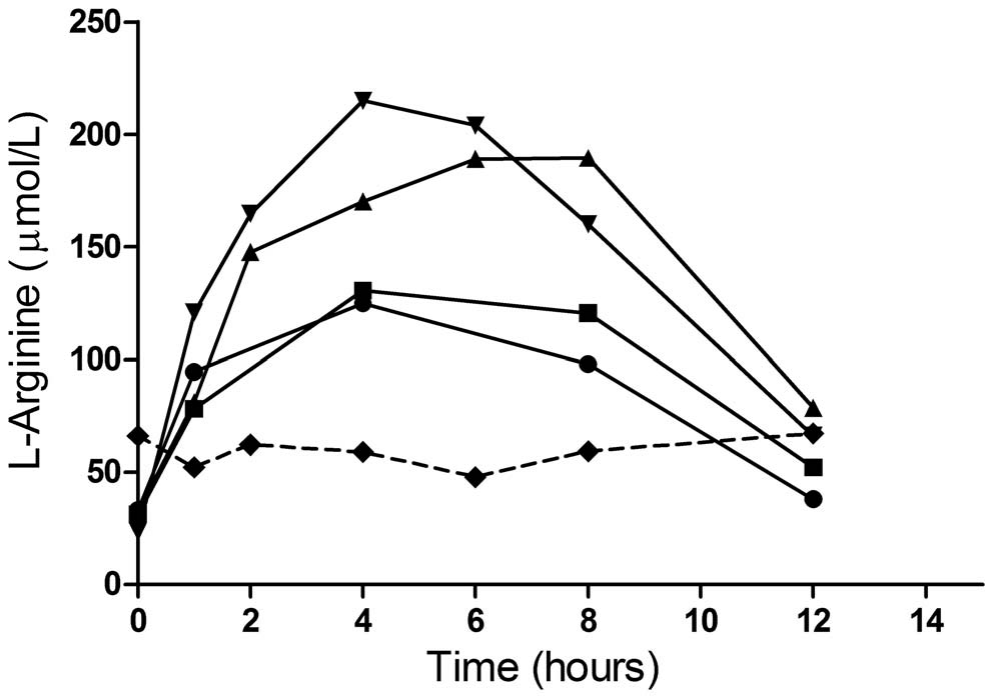
Time-concentration curves for plasma L-arginine concentrations before and after infusion of 12 g L-arginine (solid line) or saline (broken line) for patients 1 to 5. X-axis = time in hours, y-axis = L-arginine concentration in mg/L.

National changes in the implementation of pre-existing materials transfer agreements pertaining to sample export meant that serial measures of L-arginine could only be measured in the first five of the eight patients enrolled (four L-arginine and one saline). These results represent a preliminary assessment of the pharmacokinetic behavior of L-arginine in patients with SM and the time-concentration curves for plasma L-arginine concentrations are shown in **Figure 5**. In these analyses we used the same 2-compartment structural model with a polynomial model for placebo (patients who did not receive arginine) as was used in the previous population pharmacokinetic analysis of patients with moderately severe malaria [15]. For this preliminary data set the values of peripheral volume and intercompartmental clearance were fixed as their values could not be estimated based on the data from five patients. The fixed effects values of the change from baseline of L-arginine in the patient who received only saline were estimated based on the model from MSM and the parameter values were found to be similar to those for MSM. This model provided an acceptable description of the L-arginine data, based on individual plots of predicted and observed data versus time, from the 5 SM patients. From data from the 4 patients who received L-arginine infusion, the between subject variance (BSV) was able to be estimated for clearance (CL) only. The data would not support consideration of BSV terms on other parameters (such as baseline and V1). Pharmacokinetic parameters derived from the severe malaria patients are presented in table 3 and compared with those previously derived in moderately severe malaria. There were too few patients in this study to consider the influence of covariates.

**Table 3:** Pharmacokinetic parameter values estimated in the patients with severe malaria compared to those from moderately severe malaria [15].

Parameter	Moderately severemalaria (ref [15])	Severemalaria
Structural model parameters for L-arginine		
CL (L/h)	31	61.8
V1 (L)	27	115
V2 (L)	21	70.9 (fixed)
Q (L/h)	74	21.4 (fixed)
Structural model parameters for natural recovery of L-arginine		
Base	33	5.88
Gradient 1	0.0612	0.153
Gradient 2	−0.139	−0.492
Variability between patients		
BSV CL (%)	0.274	0.116
BSV V1 (%)	0.0103	0.0103 (fixed)
BSV BL (%)	0.163	0.163 (fixed)
Residual variability		
σ (proportional)	28.6%	22.5%

The moderate severe malaria parameter estimates are derived from the best model without considering covariates (for comparison with the current parameter estimates).

Baseline = base+gradient(1)*time+gradient(2)*time ^2^. Where *time* represents time post-start of L-arginine infusion.

CL = clearance, V1 = central volume of distribution, V2 = peripheral volume of distribution, Q = intercompartmental clearance, BSV = between subject variability which was assumed to be log-normally distribution and expressed as a %, σ = residual variability (standard deviation).

## Discussion

This is the first clinical trial of an adjunctive treatment aimed at increasing NO bioavailability in severe malaria. In this study L-arginine was given intravenously to adults with severe falciparum malaria at the dose of 12 g over 8 hours. There were no clinically significant adverse effects on symptoms, vital signs, selected biochemical measures and acid base status. No improvements in lactate clearance time or endothelial function were noted, though only 4 of 6 patients who received L-arginine had abnormal baseline values for lactate and endothelial function. Pharmacokinetic analysis of L-arginine demonstrated the clearance was approximately 40% higher in severe malaria, with concentrations lower than those predicted from a previous model developed in moderately severe malaria [15]. However, the small numbers of patients in each group do not permit us to make definite conclusions.

The effects of L-arginine in decreasing blood pressure are thought to be due to increased endothelial nitric oxide (NO) production [18]. The lack of decrease in mean blood pressure with L-arginine infused at 1.5 g/hour contrasted with the findings from our previous study in MSM where 12 g was given at a rate of 24 g/hour. In that study, a decrease in the mean systolic and diastolic pressure was noted towards the end of the 12 g but not the 3 g or 6 g infusions. However, our results are in agreement with a study of adult sepsis given L-arginine at 1.1 g/hour for 3 days [19], where no hemodynamic effects were reported. In contrast to the lack of change in mean systolic BP, there was a transient but statistically significant change in mean maximal decrease in systolic and diastolic blood pressure following L-arginine infusion. Both were highly correlated with the L-arginine dose/body weight ratio, suggesting a transient dose-related increase in NO production.

Consistent with our previous study, there was no statistically significant change in the mean plasma potassium concentrations, but a transient and statistically significant but clinically unimportant mean maximal increase in potassium concentration in patients given L-arginine compared to pre-infusion levels. The mean maximal ranges of increase were 0.2 to 0.7 meq/L and no patient had a potassium concentration higher than 5 meq/L, including the patients with acute renal failure. This was comparable to increases previously seen in patients with moderately-severe malaria given 6 g over 30 minutes but lower than those given 12 g in the same time period [14]. The increase is thought to result from increased L-arginine uptake by the cells with subsequent movement of intracellular potassium to the extracellular space [20]. There was also a statistically significant but clinically unimportant mean maximal decrease in glucose concentration, but again not when mean change was evaluated. These changes were again consistent with those seen in our previous study and were not close to the range where replacement is required. The lack of a clear dose relationship between L-arginine for both the maximal increase in potassium and the decrease in glucose may also indicate that L-arginine was not causal and that other mechanisms may be involved.

The only commercially available intravenous L-arginine formulation is the hydrochloride form which contains 4.75 meq of hydrogen and chloride ions per gram. In MSM patients, there was a dose-dependent relationship between L-arginine with decreases in bicarbonate and pH, and an increase in chloride [14]. In this pilot study, patients with metabolic acidosis with bicarbonate concentrations of less than 15 meq/L were excluded. There was a transient statistically significant decrease in bicarbonate concentrations in the maximal but not mean change following L-arginine infusion. There was also a significant but clinically unimportant mean maximal increase in chloride of 2.8 mmol/L compared to baseline, although this was not different to the 2.5 mmol/L change seen in those given saline. The lack of change in the pH and lactate suggest that the effect on overall acid base status was clinically unimportant. The stable anion gap and increase in chloride suggests the bicarbonate decrease was due to hydrogen and chloride contained in the L-arginine. Other agents used to increase NO bioavailability such as inhaled NO, can increase methemoglobin [21]. No increase in methemoglobin was seen following L-arginine infusion.

We did not find any improvement in the lactate clearance time, and in contrast to our study in MSM, there was no increase in endothelial NO production [8]. Previously, L-arginine was given in MSM over 30 minutes in ascending doses at rates of 6 g/hour to 24 g/hour compared to the 1.5 g/hour rate in this study in severe malaria. Simulations from a pharmacokinetic model of L-arginine infusion developed in MSM predicted that the dose used in the current study would deliver L-arginine concentrations above the *Km* of the CAT-1 transporter in 70% of the patients [15]. However, pharmacokinetic analysis in SM showed that the concentrations achieved in the current study were 40 percent lower than expected. This may be due to the increased plasma arginase concentrations found in SM compared to MSM [8], and may have contributed to the lack of endothelial response. Other factors could play a role in reducing vascular NO bioavailability in SM compared to MSM, including increased concentrations of the endogenous NOS inhibitor asymmetrical dimethylarginine (ADMA) [12], and increased NO quenching by cell-free hemoglobin [11]. The adequacy of concentrations of the NOS cofactor, tetrahydrobiopterin, in severe malaria also requires further investigation. Our results suggest that future studies of L-arginine in SM will require an increase in the dosage.

The structural model derived from moderately severe malaria appears to describe the data in severe malaria accurately. Although the small numbers of patients in whom pharmacokinetic assessment was possible limited the ability to determine accurate estimates of pharmacokinetic parameters, typical population values of CL and V appeared to be ∼40% and ∼400% larger for patients with severe malaria compared to the previous study in MSM [15]. Limitations in available subjects may have led to potential bias in the values of the parameters. It is likely that dose adjustment may be needed in patients with SM compared to MSM.

In summary, a 12 g dose of L-arginine infused at 1.5 g/hour for 8 hours in patients with SM was safe, well tolerated and resulted in statistically significant but transient and clinically insignificant effects on mean maximal change in systolic blood pressure, potassium, glucose, bicarbonate and chloride compared to pre-infusion levels. Although numbers were small and there were only modest baseline abnormalities in lactate concentrations and endothelial function, no improvement in lactate clearance or endothelial function were seen. Notably plasma L-arginine concentrations following infusion appeared to be 40 percent lower than predicted. Future studies in SM will require an increase in the dosage of L-arginine.

## Supporting Information

Protocol S1 **Trial Protocol.**
(DOC)

Checklist S1 **CONSORT Checklist.**
(DOC)
