# Supplementary material for: A Randomized Pilot Study of L-Arginine Infusion in Severe Falciparum Malaria: Preliminary Safety, Efficacy and Pharmacokinetics
Source: PLoS One. 2013 Jul 29;8(7):e69587. doi: 10.1371/journal.pone.0069587 (PMC3726665; doi:10.1371/journal.pone.0069587)
Supplement: Protocol S1 — Trial Protocol. (DOC) [file pone.0069587.s001.doc]

**Safety and preliminary efficacy, pharmacokinetics, pharmacodynamics of L-arginine in severe falciparum malaria (incorporating comparative substudies of near infrared resonance spectroscopy):**

**May 2007**

**Investigators:**

**Dra. Retno Gitawati** MS, Apt. (Research Pharmacist, Biomedical, Pharmaceutical and Research and Development Center, NIHRD): Indonesian PI

**Prof Nick Anstey** MBBS PhD (Infectious Diseases Physician, Menzies School of Health Research [MSHR]) Australian PI

**Dr Tsin Yeo** MBBS (Infectious Diseases Physician and PhD student, MSHR)

**Prof Steve Duffull** PhD (Chair, School of Pharmacy, University of Otago, New Zealand)

**Dra Indri Rooslamiati** (Research Pharmacist and Masters Candidate, Biomedical, Pharmaceutical Research and Development Center, NIHRD)

**Dra Ervi Salwati** MSc **(**Researcher, Biomedical, Pharmaceutical Research and Development Center, NIHRD)

**Dr Emiliana Tjitra** MD PhD (Senior Researcher, Biomedical, Pharmaceutical Research and Development Center, NIHRD)

**Dr Enny Kenangalem** MD (Research Clinician, NIHRD-MSHR Research Program, Timika and Dinas Kesehatan, Timika),

**Dr Ric Price** MD (Senior Lecturer, Menzies School of Health Research)

**Dr Daniel Adrian Lampah** MD (Research Clinician, Dinas Kesehatan, Timika)

**Prof David Celermajer** MBBS PhD (Professor of Cardiology, University of Sydney)

**Dr Yvette McNeil** PhD (HPLC Scientist, MSHR)

**Ms Christabelle Darcy** BSc (HPLC Scientist and postgraduate student, MSHR)

**Consultants:**

**Prof D Granger** MD**, Dr B Lopansri** MD and **Prof B Weinberg** MD (Utah and Duke Universities): HPLC and NO biology

**Prof Gareth Turner** MD (Oxford): endothelial inflammation

**Dr Chris Engwerda,** PhD (QIMR): immunology

**Dr Cliburn Chan** MBBS PhD (Duke University): computational biology

**Safety Monitoring Committee (SMC):**

**Dr Peter Morris** MBBS PhD (Clinical Trials Researcher, MSHR): Chair

**Dra. Nani Sukasediati** MS, Apt. (Senior Pharmacist, Pharmaceutical and Traditional Drug Research and Development Center, NIHRD)

**Dr Paulus Sugiarto** MD (Clinical Director, RSMM, Timika)

**Dr Allen Cheng** MBBS PhD (Clinical researcher, MSHR)

**Mr Joseph McConnell** MSc (Biostatistician, MSHR)

**Table of Contents Pages**

i. Executive Summary 3-4

1. Background 4-7

2. Study overview with flow diagram 7-9

3. Study design 10

4. Sample size calculation 10-11

5. Patient randomization 11

6. Study site 11

7. Patient entry criteria 12

9. Analysis plan 12-13

10. Outcome measures 14-18

11. Substudy 1 and 2 18

11. Safety measures 18-22

12 Time lines 22

13. Ethical issues 24-25

14. References 26-28

15. Summary table of clinical, laboratory and physiological tests 29-30

16. Appendix 1-Abbreviations

17. Appendix 2-Patient entry criteria for NIRS substudy

18. Appendix 3-Information and consent forms

19. Appendix 4-SOP for specific assays of specimens with Material Transfer Agreement

**Executive summary of studies proposed in this application:**

**Stage 3: Phase 2A and Phase 2B**

**Background:** Mortality from severe malaria remains ~15% despite the use of the most rapidly parasiticidal antimalarial therapy, artesunate. Adjunctive treatments may improve outcome. Our overall goal is to determine if adjunctive treatment with L-arginine is safe and improves outcomes in severe malaria. In stage 1 and stage 2 studies to date, we have shown that L-arginine is safe in moderately severe malaria, increases nitric oxide production and improves endothelial function. We now propose to extend these studies to patients with severe malaria.

**Aims:** To determine the safety, preliminary efficacy, pharmacokinetics and pharmacodynamics of L-arginine infusion in severe malaria.

**Methods:** Based on the pharmacokinetic modeling and simulation results of Stage 1 and 2, we propose a **phase 2A** randomised controlled trial of L-arginine vs saline in severe malaria, each given over 8 hours. If safety is demonstrated this will be followed by a **phase 2B** open-label study of 24-hour infusion of L-arginine in severe malaria with safety and preliminary efficacy compared with the 8 hour infusions given in phase 2A.

The primary outcomes will be the improvement in endothelial function and lactate clearance time in patients given L-arginine infusion compared with those who received saline. Among the secondary outcomes will be safety and the effect of L-arginine vs saline on tissue oxygen delivery (NIRS).

Data from both phase 2A and 2B will be used to generate a pharmacokinetic/ pharmacodynamic model.

**Expected Results:** L-arginine would improve the endothelial function, lactate clearance time and tissue oxygen delivery compared to saline with no clinically significant adverse effects.

**Stage 3: Substudy 1 and 2**

**Background:** Outcome measures in phase 2A and 2B studies will be the effect of L-arginine on endothelial function (EndoPAT; primary outcome) and microvascular tissue oxgen delivery measured by near infrared resonance spectroscopy (NIRS; secondary outcome). As NIRS has not been used previously to evaluate tissue oxygen delivery in malaria, the protocol therefore includes two substudies to further characterize NIRS in severe malaria and the relationship to endothelial function: the first in adults and the second in children.

Substudy 1 will compare NIRS and endothelial function among adults with severe and moderately severe malaria, sepsis and healthy controls, both at baseline and sequentially. If L-arginine is effective in adults, we plan in the future to extend the use of L-arginine as an adjunctive agent to children with severe malaria in the future. In substudy 2 we will compare NIRS and endothelial function among children ≥4 years with cerebral malaria, moderately severe malaria and healthy controls at baseline and sequentially and relate these results with plasma L-arginine concentrations.

**Aim**

**Substudy 1:** To measure endothelial function and tissue oxygen delivery in adult patients with moderately severe malaria, sepsis and healthy controls.

**Substudy 2:** To measure endothelial function and tissue oxygen delivery in children≥4 years old with severe and moderately severe malaria and healthy controls.

**Methods:** Both substudy 1 and 2 will be observational longitudinal studies.

**Expected Results:** Endothelial function and tissue oxygen delivery in patients with sepsis and severe malaria will be impaired compared to moderately severe malaria and healthy controls at baseline. The endothelial dysfunction and tissue oxygen delivery will improve in parallel with the clinical condition. There will be an association between endothelial function and tissue oxygen delivery.

**1. Background:**

Please see appendix 1 for the list of abbreviations

**1.1 Scientific background**

*1.1 How can we reduce mortality in severe malaria?*

Mortality in severe malaria remains between 15-30% despite the best available antimalarials^1^. There are two goals in designing better treatments to reduce mortality in severe malaria: 1) to kill parasites faster and 2) to decrease the excessive inflammation/tissue damage found in severe malaria. Currently there is no adjunctive therapy available which can improve the mortality rate when used in combination with anti-parasitic drugs in the treatment of severe malaria^2^.

The first goal was addressed in the joint NIHRD-MSHR SEAQUAMAT study at RSMM, Timika, part of the multi-centre SouthEast Asian trial of artesunate vs quinine (SEAQUAMAT)^3^. This demonstrated that intravenous artesunate reduced mortality of severe malaria by 35% compared with quinine. The results of this study have changed both Global WHO policy and Indonesian Ministry of Health recommendations for treatment of severe malaria from quinine to artesunate.

To address the second goal, this protocol describes the third stage of our studies that seek to determine if L-arginine can act as an anti-disease agent to dampen the excessive inflammation found in severe malaria. Stages 1 and 2 of this study have already been completed.

*1.2 Arginine and NO:* Arginine is a naturally occurring amino acid found in plant and animal protein (eg nuts). It is the critical substrate required for the generation of nitric oxide in the body by the action of a family of enzymes the nitric oxide synthases (NOS)^4^. The constitutively-expressed enzymes NOS1 and NOS3 produce low-level homeostatic neuronal and endothelial NO, whereas in inflammatory states inducible NOS (NOS2) generates high level NO production in a variety of cell types^4^, including peripheral blood mononuclear cells (PBMCs)^5^. NO has antimicrobial and immunomodulatory actions during inflammation^5-7^. Generation of NO from intracellular NOS is critically dependent upon transport of extracellular arginine into cells via the cationic amino acid transporters, CAT1 and CAT2, with the K_m_ for uptake of arginine being 70-150 uM^8^. In situations of inadequate extracellular arginine availability, NOS generates superoxide instead of NO^9-11^ which increases oxidant stress and cellular damage.

*1.3Arginine: clinical pharmacology:* In healthy humans endogenous arginine is a non-essential amino acid, synthesised from citrulline, which is in turn is synthesised from enteral glutamine and glutamate^8^. However, in acute inflammation such as in sickle cell crisis^12,13^, severe burns^14^ and sepsis^15^, plasma levels of arginine are low and arginine is “conditionally essential” ^14,16^. arginine is metabolised by arginases (in hepatocytes and other cell types) to form urea and ornithine, but there is strict segregation of hepatic and plasma arginine pools and only 5% of urea production is derived from plasma arginine^8^. arginine is also renally excreted but undergoes almost complete tubular reabsorption^17^. When exogenous arginine exceeds the renal threshold for tubular reabsorption (eg in supraphysiological dosage), there is additional renal excretion^17^. The published pharmacokinetics of arginine are derived from studies of L-arginine given in large doses only to adults with normal plasma arginine concentrations, where it has been described by non-compartmental analysis^17,18^ with a half-life for oral and IV L-arginine ranging from 40-120 minutes (with variability due in part to the non-linearity).^17^ However results for stage 1 and 2 of our arginine studies in moderately severe malaria show that the current best pharmacokinetic model in an acute illness such as malaria is a 2 compartment first order elimination model. This model has two half-lives of disposition, t½ (α) and t½ (β) with values of 15 minutes and 3.75 hours, respectively (Appendix 3).

*1.4 Nitric oxide and severe malaria:* Our studies over the last decade have associated NO production with protection from severe malaria. In African children we found an inverse association between malaria disease severity and systemic NO production/monocyte NOS2 expression^19^. In longitudinal studies, blood monocytes from children with past severe malaria had less NOS activity than those from children with past uncomplicated malaria^20^. We have also found novel NOS2 polymorphisms associated with increased NO production and protection from severe malaria^21^. In Papua, we have now recruited over 600 adults with and without severe malaria and shown the same inverse association between disease-severity and NO/PBMC NOS2 in *non-immune* *adults* from *different ethnic groups* in a region of *very different malaria epidemiology* (^22^and manuscript in preparation). Potential mechanisms for these associations include the ability of NO to decrease pro-inflammatory cytokines^23^, endothelial cell adhesion molecule expression^24^, and parasite cytoadherence (our unpublished data)^21^. In complementary immunohistochemical studies, other investigators have shown that in contrast to suppression of PBMC NOS expression/systemic NO production, tissue NOS expression is increased in severe malaria^25^. These tissue studies are unable to directly quantitate NO production. We hypothesise that inadequate arginine levels found in severe malaria limits tissue and monocyte NO production; and that by generating superoxide NOS exacerbates deleterious oxidant stress in severe malaria.

*1.5 Results from Stage 1 and Stage 2*

The key findings from stage 1 and 2 are summarized below:

*Stage 1: Natural history study: time course of recovery of hypoargininemia (and pharmacodynamic measures) in uncomplicated and severe malaria.*

The L-arginine levels were significantly lower in patients with moderately severe and severe malaria compared to healthy controls. Endothelial function and pulmonary NO, real time NO production in two different organ systems, were significantly lower in severe malaria than in moderately severe disease and healthy controls. Endothelial dysfunction correlated significantly with elevated levels of plasma soluble inter-cellular adhesion molecule 1 (ICAM-1, a known marker of endothelial inflammation) and blood lactate, a biomarker of disease severity in severe malaria and a marker of tissue hypoxia. These findings in combination suggest that low L-arginine concentrations in severe malaria impair endothelial NO production resulting in endothelial dysfunction and inflammation. We hypothesise that this results in increased sequestration by the malaria parasite to the endothelium and micro-circulatory impairment of oxygen delivery to cells resulting in organ damage.

*Stage 2: A pilot safety, pharmacokinetic and pharmacodynamic study of exogenous L-arginine in moderately severe malaria.*

In stage 2, a single ascending dose study, 30 patients with moderately severe malaria were given intravenous L-arginine at 3g (n=10), 6g (n=10) and 12g (n=10) over 30 mins.. The L-arginine infusions were all well tolerated with no significant infusion-related symptoms or biochemical side effects. In addition there was an improvement in both endothelial function and the production of pulmonary NO in the group given L-arginine compared to placebo. In patients with moderately severe disease, not all patients had endothelial dysfunction as measured by pre established criteria. However in the subgroup of patients with endothelial dysfunction as defined *a priori,* the improvement in endothelial function showed a dose response with improvement increasing with increasing amount of L-arginine infused.

This pilot study demonstrated proof of concept that L-arginine infusion in moderately severe malaria was safe and able to improve endothelial function and pulmonary NO production probably by increasing the amount of arginine available to the CAT transporters and NOS enzymes.

*1.6 Pharmacokinetic model developed from stage 1 and stage 2:*

Using results of L-arginine concentrations from stage 1 and stage 2, a pharmacokinetic (PK) model has been developed using the NONMEM software package. Summarizing the results, a 2 compartment model with first order elimination was the best model. This model has 2 half-lives of 15 minutes and 3.75 hours. The volume of clearance is 44.8 L/h, and the 2 volumes of distribution were 24.6L and 70.9L.

**2. Rationale for extending the study of exogenous L-arginine to patients with severe malaria:**

L-arginine and NO have been associated with protection in severe malaria. We have shown that several real time physiological markers of NO production (endothelial function and exhaled NO) are markedly impaired in severe malaria compared with moderately severe malaria and healthy controls. In addition results from stage 2 demonstrate L-arginine infusion in patients with moderately severe malaria is safe, improves endothelial function and increases NO production. Based on these findings, the study should be extended to patients with severe malaria as this is the patient population with the most marked impairment of endothelial function and the population in which this adjunctive therapy would potentially be used if shown to be efficacious.

**3.** **Overview of studies proposed in this application:**

*3.1* *A phase 2A randomised controlled trial of L-arginine in severe malaria*:

We will determine the safety, pharmacokinetics, pharmacodynamics and preliminary efficacy of an 8 hour infusion of L-arginine in severe malaria, given as two ascending doses.

*Hypotheses*: L-arginine infusion over 8 hours in severe malaria is safe, and will improve endothelial and microvascular function, tissue oxygen delivery and biomarkers of efficacy. The effect will be dose dependent.

*3.2* *A phase 2B open-label study of 24-hour infusion of L-arginine in severe malaria*:

To compare the safety and preliminary efficacy compared with the 8 hour infusions in phase 2A.

*Hypothesis*: L-arginine infusion over 24 hours in severe malaria is safe, and will improve endothelial and microvascular function, tissue oxygen delivery and biomarkers of efficacy to a greater extent than when given over 8 hours

*3.3* *Prospective longitudinal study of endothelial function and microvascular oxygen delivery in severe malaria:*

The outcome measures in the phase 2A and 2B studies will be the effect of L-arginine on endothelial function (EndoPAT; primary outcome) and microvascular oxygen delivery measured by near infrared resonance spectroscopy (NIRS; secondary outcome). We will therefore undertake two substudies to further characterize NIRS in severe malaria, and its relationship with endothelial function: the first in adults and the second in children.

*Hypothesis:* In severe malaria, endothelial function will be associated with microvascular obstruction, tissue oxygen delivery and blood lactate, with each measure being impaired in proportion to disease severity.

The following flow diagram outlines all the components of the **Stage 3 Arginine Infusion in Severe Malaria** study.

Severe malaria

Patients

Phase 2a: Block 1

12 patients to receive

L-arginine (12G/8hrs)

6 patients to receive saline infusion

If safety is demonstrated in Block 1, study will proceed to Block 2.

Phase 2a: Block 2

12 patients to receive L-arginine (24G/8hrs)

6 patients to receive saline infusion

NIRS Substudy 1

Adults

Sepsis patients

(n=36)

Moderately severe

malaria (n=36)

Healthy

controls (n=36)

If safety and efficacy demonstrated in phase 2A,

Phase 2B

24 patients

L-arginine 1.5-3G/hr for 24 hours

Stage 3:

Arginine infusion in severe malaria

study

NIRS Substudy 2

Children

Cerebral

Malaria

(n=25)

Moderately

severe

malaria

(n=25)

Healthy

Controls

(n=25)

**4. Study Design of Stage 3: A phase 2A randomised open-label study of L-arginine infusion in severe malaria:**

This will be a randomized open label study of L-arginine infusion in patients with severe malaria, given as two ascending doses over 8 hours.

*Treatment Allocation*: Patients with severe malaria will be randomized in two blocks of 18. The first block of 18 patients will receive either 12 g L-arginine or saline placebo. If safety is demonstrated in the first block, a further 18 patients will be enrolled in the second block and randomized to receive either 24g arginine or saline placebo

Block 1 (n=18):

1. Standard RSMM antimalarial drug regimen for severe falciparum malaria (currently artesunate) plus 12g of L-arginine diluted to a 10% solution and given over 8 hours (n=12); OR
2. Standard RSMM antimalarial drug regimen for severe falciparum malaria (currently artesunate) plus saline placebo, 240 ml given over 8 hours (n=6).

then, if safety is demonstrated in the first block

Block 2 (n=18)

1. Standard RSMM antimalarial drug regimen for severe falciparum malaria (currently artesunate) plus a 24g dose of L-arginine diluted to a 10% solution given over 8 hours (n=12); OR
2. Standard RSMM antimalarial drug regimen for severe falciparum malaria (currently artesunate) plus saline placebo, 240ml given over 8 hr (n=6).

The initial dosage of 12g was chosen based on the PK model developed from stage 1 and stage 2, and was shown to be safe in moderately severe malaria (See Appendix 3 for full PK report). The 24 g dose will test whether higher plasma L-arginine concentrations achieve greater effect with equivalent safety. Patients in the second block will receive either 24g L-arginine or saline placebo. If safety is demonstrated with 12g in the first block, however and results suggest that the higher dose of 24g may be unsafe, the 12g dose will be continued in the second block.

After completion of the 8 hour infusion of either L-arginine or saline, all patients in phase 2A will receive 15ml/hr of saline from 8-24 hours. This will ensure comparability with the groups in phase 2B receiving the 24 hour infusion of L-arginine.

Intravenous L-arginine hydrochloride is prepared as an inexpensive generic commercially available TGA-registered drug (Pharmalab, NSW, Australia ).

**5. Sample Size and Power Calculations:**

The study will not be powered to detect a difference in mortality. The physiological measure of endothelial function is a primary endpoint. Results from stage 1 showed a mean endothelial function of 1.41 in patients with severe malaria vs a normal value of 1.67 quoted by the manufacturers and mean of 1.93 seen in healthy controls. The standard deviation in endothelial function patients with severe malaria from stage 1 was 0.25.

The patients receiving any L-arginine will be compared to the group receiving saline. Patients with severe malaria will be randomized in a 2:1 ratio to receive L-arginine versus normal saline. Using a two sided significance level of 0.05 with a power of 80% to detect a significant improvement of 0.2 (a 20% difference) in the endothelial function between the groups would require approximately 20 patients in the L-arginine group and 8 in the saline group (n=28). All calculation were done with the Power and Sample Size (PS) Software Program^26^.

Based on the above calculations, we would plan to recruit 36 patients with severe malaria for phase 2A, 24 of which will receive L-arginine.

**Phase 2B:** A total of 24 patients will L-arginine over 8 hours in phase 2A. To evaluate any additional benefits of a longer infusion, in phase 2b we will therefore enroll the same number of patients for L-arginine infusion over 8 hours in phase 2A.

**Substudies:** A total of 36 patients with severe malaria will be enrolled in Phase 2A. In substudy 1, the NIRS reading in these patients will therefore be compared to an equivalent number of patients with moderately severe malaria, sepsis and healthy controls. To detect a difference in StO2 values between these patients and those with severe malaria, the planned number of 36 in each group will be enough to detect a biologically significant difference based on previous studies in patients with sepsis^27^. Based on the findings in adults (stage 1), a sample size of 25 in each group should be sufficient to demonstrate a difference in EndoPAT among children with cerebral malaria, uncomplicated malaria and healthy controls.

**6. Patient numbering, randomization and blinding:**

Once the patient has a confirmed diagnosis of severe falciparum malaria, has fulfilled all the inclusion criteria, has none of the exclusion criteria. Written informed consent from either the patient or the patient’s relative will be obtained to participate in the study, and he/she will be allocated the next code of the study. Each code has a sealed opaque envelope which will contain that patients’ treatment group and which will only be opened when a patient has been allocated a code number.

All patients screened for enrolment will be entered into a logbook. Those patients screened but not enrolled will have the reason for non-enrolment documented.

Clinical care, including antimalarial drug therapy and supportive care will be undertaken by the treating RSMM doctor. The Research clinician will not be blinded to treatment allocation. The patient and the research nurses and doctor undertaking the clinical and physiological measures below will be blinded to treatment allocation, as will the laboratory scientists performing the assays.

**7. Study Site:**

Patients will be enrolled at RS Mitra Masyarakat (RSMM), Timika, Papua, Indonesia. A malaria research collaboration is already established with this hospital as part of the MOU between NIHRD, RSMM, Regional Ministry of Health and MSHR. It is the site of our recent ACT trials, ACT cost-effectiveness, arginine trial, NO-malaria studies, and SEAQUAMAT malaria studies all of which have involved capacity building and clinical research training to local investigators.

RSMM has approximately 200 malaria admissions per month. The infusions will be done in the high dependency unit (HDU) where the patients will be monitored for the duration of the infusion or longer if deemed necessary by the treating physicians.

**8. Patient entry criteria:**

*Severe malaria (SM)*, defined by modified WHO criteria^28^:

Inclusion criteria:

1. age 18-60 years
2. informed consent obtained
3. time of commencement of artesunate ≤18 hrs before infusion of L-arginine
4. any level of *P. falciparum* parasitemia, and one or more of the following criteria:
   1. acute renal failure (creatinine >265umol/L)
   2. hyperbilirubinemia (total bilirubin >50 umol/L) with either renal impairment (creatinine >130umol/L) or parasitemia of >100,000 parasites/uL
   3. blackwater fever
   4. hyperparasitemia (>10% parasitised red cells)
   5. cerebral malaria (Glasgow coma score <11)
   6. Hypoglycemia
   7. Respiratory distress (RR >32)

Exclusion criteria:

1. pregnancy or lactation
2. diabetes
3. serious pre-existing disease (cardiac, hepatic, kidney)
4. systolic blood pressure <90 mmHg after fluid resuscitation
5. initial iSTAT test showing any of the following values:
   - 1. K+ > 5.5 meq/L
     2. Cl- > 110 meq/L
     3. HCO3- < 15 meq/L
6. known allergy to L-arginine
7. evidence of concurrent bacterial infection
8. concurrent therapy with any of the following medications:
   - 1. spironolactone,
     2. oral nitrates,
     3. phosphodiesterase inhibitor (eg sildenafil [Viagra])
     4. alpha-blocking antihypertensive agents (eg prazosin)
     5. L-arginine

All subjects with SM will be treated with artesunate as per standard RSMM protocols and will commence L-arginine within 18 hours of commencing artesunate. Height and weight will be documented in all subjects.

**9. Analysis**

**9.1 Primary outcome measures:** in severe malaria patients who receive L-arginine infusion versus those receiving saline infusion:

1. **Improvement in endothelial function.** Change in endothelial function after the completion of the 8 hour infusion
2. **Lactate clearance:** area under the curve until lactate returns to the upper limit of normal.

**9.2 Secondary outcome measures**:

1. Safety. Clinical and biochemical measures (see below). In those receiving L-arginine, biochemical and hemodynamic measures at the completion of infusion will also be compared with measures at the start of infusion.
2. Change in endothelial function (1 hour response and end of infusion response) in each arginine infusion regimen vs saline placebo combined
3. Paired change in endothelial function paired comparison of post-vs pre-infusion values, overall, and in each arginine infusion regimen
4. Lactate clearance for each infusion regimen
5. Fever clearance time
6. Parasite clearance time
7. Change in L-arginine concentration
8. Improvement in microvascular obstruction (OPS)
9. Tissue oxygen consumption (NIRS)
10. Tissue oxygen delivery (NIRS)
11. Change in NO production
12. Change in oxidant stress
13. Change in red cell deformability

We will also utilize clinical, physiological and biochemical data from the phase 2A and 2b studies to generate a pharmacokinetic model of the L-arginine concentration-time profile using NONMEM^29^ software to establish the population estimates, mean values and between subject variability of the following parameters: clearance, volume of distribution and half life. A pharmacodynamic model will also be developed and fitted to the data relating L-arginine plasma levels with the physiological measures such as endothelial function, StO2, exhaled NO and microvascular obstruction.

The two will be combined in a PK/PD model which will relate the dose of L-arginine given to their plasma concentration as well as their effects on the physiological measures.

We will also develop a model to predict endothelial and microvascular function in malaria.

**9.3. Independent blinded statistical analysis** will be performed by the DSMC statistician at the completion of the 36 enrolments in phase 2A to assess the statistical significance of the two primary endpoints, and if not significant, the estimated sample size that would be required to achieve significance for the two endpoints.

If significance for the two endpoints is not achieved, and the calculated additional sample size is deemed feasible and acceptable by the independent statistician and the research team, then the study can be continued to achieve the revised sample size, subject to approval by the Data Safety Monitoring Committee.

The DSMC will also assess safety of administration in phase 2A.

**10. Phase 2b study**

If results from stage 2A demonstrate safety and evidence of efficacy (from at least one primary or secondary endpoint) when administered over 8 hours, the study will proceed (after agreement of the DSMC), to an open-label phase 2b study of L-arginine infusion over 20 hours in an additional 24 patients with severe malaria (defined as in phase 2A). This will assess whether a more prolonged infusion will give additional benefit over the 8 hour infusion used in phase 2A, and that a more prolonged infusion will be safe.

The proposed dose will be 1.5g/hour for 24 hours (36g total). Should the phase 2A dose of 3g/hour be safe and show evidence of additional efficacy over 1.5g/hour, the DSMC will consider a proposal from the investigators for a dose of up to 3g per hour. The analysis will compare the effect of the 24 hour vs 8 hour infusions on each of the pimary and secondary endpoints used in phase 2A.

The same clinical, biochemical and physiological measurements used in phase 2A will also be used in phase 2B. The total blood volume and the total number of blood draws to be taken in phase 2B will be the same as in phase 2A.

Results from phase 2A and 2B will be modeled in a combined PK-PD model.

**11. Measures of safety and efficacy to be used in this study:**

***11.1 Clinical:*** Vital signs (pulse rate, BP, temperature, respiration rate) and a standardized clinical assessment (symptoms/sign list) will be serially monitored over at least 48 hours, with further measurements continuing if the patient remains hospitalised. ECG monitoring will be done from 5 minutes before infusion to two hours into infusion (at which time steady state levels will have been achieved). Co-oximetry will provide a continuous reading of pulse rate and oxygenation

***11.2 Electrolytes and Biomarkers of outcome:***

*11.2.1 Blood sampling protocol:*

Procedures used successfully in stage 2 in moderately severe malaria will be used. Two venous cannulae will be used:

A cannula in one forearm will be used for administration of IV L-arginine, and for any IV fluids required after L-arginine administration.

A cannula in the other forearm or antecubital fossa for sampling of venous blood and for any IV fluids required during arginine infusion.

At the time of cannula insertion we will take 6 ml heparinised baseline blood. This will be the baseline sample: 60ul will be used for baseline iSTAT EC8+ measurement of electrolytes, glucose, urea, and bicarbonate. The remainder will be processed as previously done in Stage 1 and Stage 2 for heparinised blood.

Subsequent sampling regimen (from cannula): up to 23 ml (in heparin) over the next 72 hours, taken in up to 12 divided aliquots during this time interval. Including the enrolment sample, this amounts to a maximum of 29ml taken over 72 hours (see table 1 for the example of the sampling regimen). For the pharmacokinetic-pharmacodynamic modeling it is advantageous for serial blood samples/measures to be collected at varied time points. The initial samplings are required to monitor safety (electrolytes) and to model the PK of L-arginine infusion in severe malaria; later samplings are required to model the return of plasma arginine to normal values and to evaluate the impact on lactate clearance time.

Our experience from the Stage 1 study in 2005-06 was that the RSMM treating clinicians routinely used the immediate results of our quality-assured bedside iSTAT readings (electrolytes, glucose, renal function and lactate) to guide their clinical management. This frequently replaced the need for blood-taking that would otherwise have been taken for clinical purposes.

*11.2.2: Blood measurements to be undertaken:* These samples include serial measurements required for clinical management of malaria (including serial parasitemia, blood glucose, electrolytes and renal function), pharmacokinetics of L-arginine (L-arginine, its metabolites, and related amino acids), pharmacodynamic measures (NO metabolites and oxidant production, electrolytes, pH) and biomarkers of response to antimalarial treatment (including lactate, glucose and parasitemia); and safety, including serial glucose, K^+^, Cl^-^, HCO_3_^-^, pH, PO_4_^3-^, and later measurements for urea, creatinine, lactate, liver enzymes and creatine kinase (see table 1).

*Plasma* will be cryopreserved at -70ºC for later quantitation of the following:

- L-arginine, its metabolites, and related amino acids by HPLC^30^ (Shimadzu) using our established methodology. This will also include assaying the arginase enzyme involved in the metabolism of L-arginine.
- NO metabolites (nitrate plus nitrite) using Greiss reagents as we have performed previously.
- CK, LFTs, lactate, urea, creatinine and the electrolytes required for study 2 below (safety measures, and efficacy measures [lactate]).
- sICAM-1, endothelin and other biochemical measures of endothelial activation
- Measures of hemolysis and NO quenching (including cell free haemoglobin, haptoglobin and LDH)
- Cytokines, both pro-inflammatory (including IL-6, TNF-α) and anti-inflammatory (including IL-10, IL-13 and IL-17), to correlate with their effect on endothelial function and to develop a computer model of the endothelial function and oxygen delivery in malaria.

*Peripheral blood mononuclear cells* will be separated from the initial and post-infusion blood samples (6ml collections), to measure the effects of L-arginine on oxidant production by white cells and white cell function (including expression of the arginine-dependent T cell receptor CD3ζ).

***11.3 Urine*** will be collected on admission and whenever this is passed whilst in hospital for serial measures of NO and oxidant metabolites and tetrahydrobiopterins (cofactors for NO synthesis from L-arginine). Samples are not filtered or centrifuged because tetrahydrobiopterins species may be associated with particulate material in the urine^31^. Freshly obtained urine will be separated into two samples: one for NO metabolites; the other (containing DTE stabilizing agent) for tetrahydrobiopterins analysis by HPLC.

***11.4 Serial physiological measures:***

The following bedside physiological tests will be measured to evaluate the effect of L-arginine on endothelial and microvascular function. The relationship between plasma arginine and each of these physiological measures will be modeled in a PK-PD model.

**11.4.1. Tests performed previously during Stage 1 and Stage 2.**

**11.4.1.1 Endothelial Function:** This will be done using the Endopat2000 instrument which was used without problem during stage 1 and stage 2 on approximately 250 patients. This non-invasive test uses two finger probes, one placed on each index finger, to measure finger volume using a technique called peripheral arterial tonometry (PAT). This involves the patient lying supine for 15 minutes. There is a 5 minute baseline period after which a blood pressure cuff placed around the non-dominant forearm is inflated to a pressure 50mmHg above the patients systolic blood pressure to induce an ischemic stress for 5 minutes, the cuff is then released and another 5 minutes of reading are taken before the test is completed. Endothelial function is assessed by the change in PAT results before and after the ischemic stress is applied. The instrument was found to be very well tolerated by patients with no adverse effects or complaints noted during or after measurement of endothelial function.

The endothelial function testing will be done prior to and immediately after L-arginine/saline infusion. Further serial testing will be done at approximately 24 hour intervals after the initial test until patient’s discharge (see tables 1 and 2).

**11.4.1.2 Exhaled NO:**

Measurements of exhaled NO will be performed using the NiOx apparatus as used previously in Stage 1 and Stage 2 using American Thoracic Society guidelines^32^. This will involve up to 10 measurements over 24-96 hours (see table 1). In stage 1 approximately 50% of the severe malaria patients without coma were able to perform this test compared to 90% (44 of 48) of patients with moderately severe malaria. Overall the tests were well tolerated with no adverse effects in both stage 1 and stage 2.

**11.4.2 New physiological tests to be used in stage 3**

**11.4.2.1 Microcirculatory Obstruction Using Orthogonal Polarizing Spectroscopy:**

**Rationale for OPS testing**: Increased production of NO by blood vessels has been shown in vitro to reduce the number of parasitized red cells sticking to endothelial cells, the cells lining the blood vessels. We hypothesise that L-arginine will allow small blood vessels to produce more NO and to become less sticky and less obstructed.

OPS has also been used in studies on patients with sepsis and was able to show an improvement in microcirculatory flow after infusion of nitroglycerin, a medication that also increases NO.^33^

**Methodology:** We propose to examine whether L-arginine can reduce the obstruction of the very small blood vessels by parasitised red cells in patients with severe malaria. To do this we also intend to directly observe the microcirculation using an instrument called a orthogonal polarizing spectroscope (OPS) (Microvision, Amsterdam, Holland) to directly visualize the capillaries of the rectal mucosa. In studies by the Mahidol-Bangladesh group (Dondorp et al, submitted for publication), OPS was well tolerated and was able to directly visualize and quantitate the degree of obstruction of blood vessels by the malaria parasites in patients with severe malaria (Dondorp A). The procedure involves insertion of a rectal probe 5mm in diameter (similar to a rectal thermometer) and obtaining 3 readings from 3 different areas for 20 seconds each time. In the Bangladesh study, OPS was performed on 44 patients with severe malaria and was well tolerated with no complications. Serial examinations also showed that the improvement in microvascular obstruction and sequestration occurred in parallel with the clinical improvement.

The OPS will be performed in patients with severe malaria only, at the same time interval as the endothelial function and StO2 measurements. We will use longitudinal measurements of improvement in the severe malaria and placebo group, with each patient being their own control.

The cultural sensitivity of performing this procedure in Papua has been discussed with Dr Enny Kenangalem, Papuan research clinician, who is of the opinion that it should be feasible acceptable in patients with severe malaria. Rectal thermometers are considered similarly acceptable in patients who are unwell.

**11.4.2.2 Tissue Oxygen Saturation (StO2):**

**Rationale**: We hypothesise that L-arginine will not only improve endothelial function (EndoPAT) and reduce the obstruction of small blood vessels (OPS), but will also improve blood flow through the very small blood vessels and improve oxygen delivery to the tissues. To test this hypothesis we will measure the effect of L-arginine on StO2.

**Methodology:** This test measures oxygen delivery to the tissues through very small blood vessels. During the endothelial function measurements, we will measure the StO2 of the thenar muscles using a non-invasive instrument called the Tissue Spectrometer (In-Spectra, Hutchinson Technologies, USA). This involves a painless non-invasive probe placed on the skin of the palm below the thumb. Near infrared resonance spectroscopy measures the hemoglobin oxygen saturation of blood in the thenar muscles. We will apply this probe at the same time that the endothelial function test finger probes are placed on the fingers. By measuring the rate of decline of StO2 during the ischemic stress applied during the endothelial function testing we can measure the oxygen utilization during ischemia. By measuring the recovery of StO2 after release of this stress^27^, we can measure the oxygen delivery capacity after removal of the ischemia. This will be done at the same time as the endothelial function testing and will therefore result in no additional procedures. We will determine the relationship between endothelial function testing and NIRS to determine how well endothelial function predicts tissue oxygen delivery. In parallel measurements we will also apply a painless non-invasive probe to the skin above the temporal bone to measure basal tissue oxygen saturation in the cerebral cortex. This will allow assessment of any improvement in StO2 in patients with cerebral malaria.

**Substudies (see flow chart)**

NIRS has not been measured in patients with malaria before and may prove to be a valuable tool to measure tissue oxygen delivery in severe malaria. We do not know the normal range in this population, or whether NIRS is also impaired in moderately-severe malaria.

In **substudy 1,** we intend to compare the baseline results of StO2 and endothelial function tests in the 36 patients with severe malaria enrolled in phase 2A of the L-arginine trial with 36 adult patients with moderately severe malaria, 36 adult healthy controls and 36 adult patients with sepsis (see inclusion and exclusion criteria in Appendix 5), on days 0,1 and 2, in association with 10 ml blood on day 0, 2ml on day 1 and 2 ml on day 2, for the same measurements as those in the L-arginine RCT described in 9.2.2 above. Comparing the StO2 measurements in severe malaria with sepsis patients will allow us to test whether the mechanism of impaired tissue perfusion in severe malaria is similar to that found in sepsis. While microcirculatory obstruction secondary to parasite sequestration is the major hypothesis for how malaria causes severe disease, others have hypothesized that in severe malaria there is metabolic failure due to mitochondrial dysfunction secondary to excess inflammatory cytokines causing cellular and organ damage similar to that proposed to occur in sepsis. Studies in sepsis conducted using NIRS^34,35^ show that it is well tolerated and suggestive of mitochondrial dysfunction. By comparing StO2 and endothelial function between severe malaria and sepsis we can answer this important question. All patients with sepsis will receive standard care by RSMM clinicians. All will receive ceftriaxone and gentamicin as per standard RSMM protocol.

**Substudy 2:** Severe malaria is also a major cause of death in children. There are no published data on endothelial dysfunction or NIRS in children with cerebral malaria (CM). It will also be important to know if endothelial and microvascular dysfunction is also found in children with CM, as this would indicate the potential for future adjunctive therapy targeting the endothelium in this group (if shown to be effective in our adult studies). Our preliminary data from Ghana indicate that EndoPAT testing is feasible and reliable in children ≥4 years of age: no adverse effects or significant complaints from EndoPAT were experienced. We therefore also propose to measure serial measures of endothelial function (EndoPAT) and NIRS in children aged ≥4 years of age with CM (n=25) (day 0, 1, 2) and compare results with children with moderately severe malaria (n=25) vs healthy controls (n=25). Inclusion and exclusion criteria are indicated in Appendix 5. Venous blood will be collected from the antecubital fossa (Up to 7ml on day 0, 2ml on day 1 and 2 ml on day 2), for the measures described in 9.2.2). EndoPAT and NIRS results will be compared across disease categories, with each other and with plasma arginine, and values on days 1, 2 during recovery.

**12. Potential adverse effects of L-arginine infusion in severe malaria; monitoring and rescue protocols if required.**

In Stage 2 there were 30 patients given intravenous L-arginine at dosages of 3g, 6g and 12g with no significant adverse events attributable to L-arginine. In the more clinically ill patients with severe malaria, we think that the risk of significant adverse effects is also unlikely but, as in stage 2, our protocol will allow us to detect and rectify any adverse events.

*a) Effects on blood pressure:* Patients with moderately severe malaria in stage 2 who received L-arginine had no clinically significant blood pressure changes. In other studies the effects on blood pressure are usually minimal, even with the 30g IV dose used , which is higher than the doses we used in stage 2 and will use in stage 3^17,18^. Endothelial NOS expression in severe malaria^25^ makes it important we monitor this closely. L-arginine supplementation in sepsis has not significantly exacerbated hypotension^36^ despite demonstrated tissue NOS expression in sepsis^37^. In previous studies, any effect on blood pressure has been minor and seen during the infusion, returning rapidly (within minutes) to normal as soon as the infusion is finished. To monitor for hypotension, the subject’s vital signs including blood pressure will be taken every 10-15 minutes prior to infusion. During the 8 hour infusions, vital signs will be monitored every 15 minutes for the first two hours, then 20-30 minutely for the remainder of the infusion, and for the first 1 hour after infusion, then hourly for 2 hours then 2-4 hourly thereafter.

Response: If systolic blood pressure falls >25 mmHg or below 80mmHg or if the attending clinician is clinically concerned, the L-arginine infusion will be stopped and legs elevated. Previous studies using higher doses of L-arginine than we will use in this study have shown the BP rapidly returns to normal within minutes of stopping the L-arginine infusion, and this is expected to be all that is required. If SBP persists < 80 mmHg, 0.9% saline will be infused at a rate based on the treating clinician’s clinical judgement.

*b) Electrolyte abnormalities:*

- *Effect on K+:* Hyperkalemia may result from redistribution of K^+^ from intracellular fluid to extracellular fluid. In stage 2, the maximum increase noted in any individual patient was 0.7meq/L and in the group that received the highest dose of L-arginine (12g) the mean maximum increase was 0.5meq/L which recovered to baseline levels within one hour. In stage 2, all doses were given over 30 mins compared to the 8 hr infusions proposed in stage 3. These slower infusions will make any K+ increases more modest, Subjects with baseline K^+^>5.5 meq/L will not be included in stage 3. Therefore a sustained and clinically important rise in K+ to ≥ 6.5 meq/L will be very unlikely. We will use iSTAT cartridges (which use a few microlitres of blood) at the bedside to instantly measure electrolytes (as well as glucose and renal function) prior to infusion, and at regular intervals during and after the infusions (see table 1). These serial measures will be clinically useful to the treating RSMM clinician.

Response: If serum K^+^ rises to ≥ 6.5 meq/L it will be immediately repeated using the bedside iSTAT to exclude hemolysis of sample. If K^+^ ≥ 6.5 meq/L or if significant ECG changes of hyperkalemia develop, the infusion will be stopped and subjects will be given 10ml of 10% calcium gluconate for membrane stabilization. This will be followed by continuous ECG monitoring and 10-15 minutely monitoring of K^+^: if thought necessary by the attending clinician, 10 U of regular insulin will be administered with 25g of IV glucose to induce K^+^ shift into cells.

- *Hypophosphatemia:*

In stage 2, the mean decrease in serum phosphate was 0.2 mg/dL which was clinically insignificant .A clinically insignificant transient fall in serum phosphate is noted with 30g infusions, with the maximum fall below baseline of 1.11 mg/100ml (or 33%), peaking 60 mins after commencement of a 30 min infusion^38^. In no cases did phosphate levels fall to potentially clinically significant levels (<1 mg/dL)^38^. We will nevertheless measure phosphate levels at baseline and at 1, 4, 8, 9 hours after the start of infusion (table 1).

Response: Moderate hypophosphatemia (1-2.5 mg/dL) is almost always asymptomatic and requires no therapy. Moreover, transient hypophosphatemia following L-arginine infusion *will not reflect any depletion of total body phosphate* that is required for very low levels of plasma phosphate to cause clinical manifestations. Low phosphate will therefore not be treated unless deemed essential by the treating clinician.

*c)* *Thrombophlebitis* is unusual, but has been described^39^. In stage 2 we used a ≤10% solution which was very well tolerated with no clinically apparent thrombophlebitis. Minor discomfort at the infusion site was noted in 2 of 30 subjects receiving L-arginine, but also in a number of saline controls. We will use the same procedure used in stage 2 and administer L-arginine as a 10% solution with careful insertion and observation of IV canula/symptoms/signs before/during infusion.

Response: If extravasation occurs, the infusion will be stopped, the affected extremity elevated. The catheter/needle will not be removed immediately, but rather left in place to aspirate any extravasated fluid. The line will then be removed and an ice pack applied.

*d)* *Anaphylaxis*: We are aware of only one report of a case of *anaphylaxis^40^*. This would seem a very rare event with an exogenous/endogenous amino acid and is considered extremely unlikely in current studies

Response: The L-arginine infusion will be ceased. Adrenaline and resuscitation facilities will be readily available in the high care unit at RSMM, to which the clinical research ward is attached. Standard treatment for anaphylaxis will be undertaken in the very unlikely event it would be required.

*e)* *Rash:* maculopapular rash: rare; causality not proven. Response: infusion will be stopped

*f) Potential effects on blood glucose:* L-arginine stimulates both insulin and glucagon release^41^. Because levels of glucagon increase to levels higher than that of insulin, the net effect in humans is reported to be a slight hyperglycemia^41,42^. Importantly, despite over 40 years’ of use of intravenous L-arginine in humans, there are **no** clinical case reports of hypoglycemia ever occurring after L-arginine infusion. In stage 2, in only one patient who received an L-arginine dose of 3g was there a decrease in blood glucose to 3.2mmol/L (*not* to a level defining hypoglycemia [3 mmol/L]) with the patient remaining asymptomatic. There were no significant changes in the patients who received the 6g and 12g doses and the decrease in glucose levels in the one patient was attributed to concurrent quinine therapy which is well documented to be associated with hypoglycemia. This will be less of a concern in stage 3 as the new policy at RSMM is to treat all patients with severe malaria with intravenous artesunate which does not result in insulin secretion and contribute to hypoglycemia. However, severe malaria itself can result in hypoglycemia and so patients will still require monitoring after infusion. Blood glucose will be rigorously monitored in both studies as outlined in table 1: We will use iSTAT cartridges (which use a few microlitres of blood) at the bedside to instantly measure glucose (as well as other electrolytes) prior to infusion, and at regular intervals during and after the infusions (see tables 1 and 2).

Response: If blood glucose falls below 3 mmol/L, the L-arginine infusion will be stopped and 50% dextrose will be administered as per standard RSMM/MoH practice in severe malaria, with blood glucose continuing to be monitored 15 minutely to 60 mins then 30 minutely until 480 mins.

*g) Potential effect on pH:* L-arginine hydrochloride contains H^+^ ions (4.75 mEq/g). While there is a theoretical potential for acidosis there have been no reports of this. Hertz *et al* (1972) ^43^ carefully documented the effect of IV L-arginine on arterial pH. They found **no change** in the pH despite infusion of 30g (much higher than we will use in this study) into patients with renal failure^43^. Clinically significant change in pH has not been found except in patients with hypochloremic alkalosis (where L-arginine hydrochloride corrects the Cl deficiency and returns the alkalosis toward normal).

The maximum theoretical decrease in bicarbonate in a 60kg patient given 12g of L-arginine hydrochloride which contains 57meq/L of H^+^ ions with bicarbonate space of 50% is approximately 1.9 meq/L. Using this change in bicarbonate to calculate the change in pH using the Henderson-Hasselbach equation, would be a maximum decrease of 0.03 units in the pH^44^, however respiratory compensation is likely to make this even smaller.

In stage 2, overall there was no significant decrease in plasma bicarbonate or pH after L-arginine infusion. In a single patient (only) given 12g there was a drop of 5meq/L in the bicarbonate level but there was no change in pH. In stage 3 we will be infusing L-arginine more slowly than in stage 2, allowing compensation and attenuating any change in bicarbonate. Moreover, any improvement in vascular perfusion by arginine would reduce the risk of acidosis.

We therefore do not expect any clinically significant acidosis, but will monitor serial real-time venous pH, PCO_2_, chloride and bicarbonate at the bedside using iSTAT EC8+ cartridges during and after infusion (see table 1)

Response: L-arginine infusion will be ceased if venous bicarbonate falls below 10 mmol/L and treatment with intravenous bicarbonate will be instituted only if pH falls below 7.1.

*h)* *Use in renal failure:* Two recent studies show that renal blood flow and glomerular filtration rate (the most accurate measure of renal function) improve after the infusion of L-arginine. Schlaich et al,^45^ studied patients with normal renal function and used an intravenous infusion of L-arginine at doses of 100mg/kg (7 Grams for a 70 kg adult) and 500mg/kg (35 grams for a 70 kg adult) over 30 minutes. They showed a statistically significant improvement in both renal blood flow and glomerular filtration rate at each dose. In the second study^46^, an intravenous dosage of 17 mg/kg gave the same improvement in normal subjects. Reassuringly, in patients with renal impairment in this study, L-arginine infusion was safe and did not cause any worsening of renal blood flow or glomerular filtration rate. We can find no case reports or primary clinical studies that report renal toxicity with L-arginine. We therefore do not expect any change in renal function in studies 2 and 3, but we will monitor renal function by serial iSTAT bedside biochemistry (see Table 1).

*i) Methaemoglobin:* Doses of 30g arginine/day have caused a very small and clinically insignificant rise in methaemogolobin in patients with sickle cell disease^47^. Using modern methods, significant methaemoglobinemia has not been found in acute malaria^48^. We will measure methaemoglobin and carboxyhaemoglobin hourly using non-invasive co-oximetry (Masimo, CA, USA), and confirm baseline readings at the RSMM lab using 200ul of venous blood. Infusion will be stopped if methaemoglobin exceeds 15%, well below the level when this becomes clinically significant.

*j) Minor adverse effects:* L-arginine can uncommonly cause nausea, vomiting, flushing, headache, and numbness. These will be documented in the CRF. These are also common in malaria, and will not result in cessation of the infusion, unless patient requests to withdraw from the study. Gastrointestinal side-effects (eg nausea, vomiting) have not been seen with IV infusion in SCD. Minor flushing has been seen. However, from our results in stage 2 there was no increase in symptoms in patients given L-arginine compared to those given saline.

*k) Consequences of tissue NO production from tissue NOS.* We believe that tissue NO production will have tissue-protective effects and will also reduce generation of superoxide from arginine-deficient NOS. Nevertheless we will be able to detect unexpected deleterious effects of increased tissue NO. As well as the organ-specific biochemical markers above (including creatinine, creatine kinase and liver enzymes/bilirubin), our existing lung function laboratory at RSMM and the exhaled NO apparatus will give us the unique opportunity to relate exhaled NO to non-invasive measures of pulmonary gas transfer (DLCO) on day 3, a sensitive markers of lung injury. DLCO was feasible and well tolerated on day 3 in severe malaria patients in our recent pulmonary malaria studies in Timika.

In stage 2 there were no clinically relevant adverse effects noted from increased NO production. In contrast in a clinical trial of a NOS inhibitor designed to reduce NO production in patients with sepsis, the mortality rate was increased.

**Serious adverse events:** The following adverse events will be deemed serious, and will be notified immediately to the Chief Investigators and to the Chair of the Data Monitoring Committee for immediate review:

1. Death*.
2. Serum K^+^ rising to ≥ 7.0 meq/L with ECG changes
3. Anaphylaxis
4. SBP falling below 70 mmHg unresponsive to saline infusion or dopamine/dobutamine infusion.

*NB In our recent RCT of artesunate vs quinine for severe malaria, 8-10% of patients with severe malaria died.^3^ Therefore death from severe malaria is likely in this study. All deaths in patients with severe malaria will be immediately reviewed by the chief investigator and notified to the Chair of the Data Safety Monitoring Committee.

**13. Proposed timeline (subject to ethics approval process):**

February-August 2007 Ethics review in Darwin (MSHR) and Jakarta (NIHRD)

September (once ethical clearance)-April 2008: Enrolments phase 2A

Preliminary safety review by DSMC

April 2008-September 2008: Enrolments phase 2b

September 2007- December 2008: measurements of L-arginine, and NO

September 2008: review by Data Safety Monitoring Committee and PD-PD modeling of phase 2A and 2b together and

*Ethics*

Approval for the study will be obtained from the Ethics Committee of National Institute of Health Research, Ministry of Health, Jakarta, Indonesia and the Ethics Committee of Menzies School of Health, Darwin, Australia. The study will only proceed after approval from both Committees.

**14. Ethical issues:**

*Potential side effects of L-arginine:* Patients with severe malaria have mean levels of L-arginine *below* normal, and combined with a dose below the standard 30g IV dose used in other settings, we do not expect significant adverse effects to occur. Whilst we believe adverse effects to be unlikely, our protocol outlined above allows us to detect, and if necessary rectify them.

*Standard treatment:* Regardless of whether they receive L-arginine, all patients will receive prompt standard antimalarial drug treatment based on standard Depkes and RSMM protocols, currently artesunate for severe malaria. There will be no delay in starting treatment due to participation in this study.

*Blood samples:* the maximum total of 29ml of blood taken over 72 hours is safe. Those with significant anemia (Hb ≤5 g/dL) will be excluded. Serial sampling is essential for appropriate pharmacokinetic-pharmacodynamic modeling. Part of the blood taken for this serial sampling will be used for iSTAT readings for electrolyes, glucose and renal function, all of which will be useful to the treating doctor, who will be alerted to abnormal results of clinical importance.

Serial blood sampling has the potential to cause concern to patients. In stage 1 and stage 2 we used flip charts during the consent process to illustrate visually the procedure of studies that involve blood sampling. We will use similar flip charts to demonstrate visually the total volume of blood that will be collected during his study. The flip chart will illustrate that the very small volumes collected during serial bleeds add up to only a relatively small and safe volume of blood. The use of these flip charts have proved to be especially useful in explaining blood volumes in patients with limited education who have limited numeracy and literacy skills.

*Cannula:* The consent process will explain how the cannula will be the same as that used clinically for medications and fluids. The information process will clarify that the cannula will ensure that the serial samplings will be painless and avoid the need for further painful needlesticks or fingerpricks. We will also explain that the blood taken from the cannula will also be used for any serial clinical tests requested by the treating RSMM doctor (eg repeat parasite counts, blood glucose, repeat hemoglobin), thus avoiding painful followup fingerpricks required for clinical purposes. Moreover, the serial iSTAT readings will be useful to the treating doctor, who will be alerted to abnormal results of clinical importance.

*Consent:* The Written Subject Information Statement will be translated into Bahasa Indonesia following any revisions recommended by the NIHRD ethics committees. If Bahasa Indonesia is a second language, the information will be translated with a bilingual RSMM staff member. In patients too ill to give consent, informed consent will be sought from from available relatives. In substudy 2, informed consent will be obtained from the parents/guardians of the children enrolled.

*Confidentiality:* Results will be stored electronically at RSMM and later at MSHR/NIHRD, accessible to investigators only, and password protected. Data sheets will be stored in a locked, secure location at RSMM.

*Endothelial Function Studies:* The procedure was well tolerated during stage 1 and 2. These *forearm* (*not* above elbow) measures are not painful. The subject may experience minor discomfort during inflation of pressure cuffs during the studies. This discomfort will be transient and is no worse than having blood pressure measurement in routine clinical practice.

*Exhaled NO:* The exhaled NO tests to be used in this study are the same used in stage 1 and stage 2 which were well tolerated.

*Tissue Oxygenation Content:* The will involve no extra inconvenience to the patient than that already involved in the endothelial function testing. It will involve a non-invasive probe which will be put on the thumb muscles of the patient at the same time they have two finger tip probes placed during endothelial function testing.

*Microvascular Obstruction:* The rectal probe used in this instrument is no larger than a rectal thermometer used in routinely in the clinical care of critically ill patients. In addition the total time that the test would involve would be a total of 60 seconds again similar to the time a rectal thermometer would be used. This test will only be done on patients with severe malaria.

*Funding:* Funding is from the Wellcome Trust (a non-profit UK-based medical charity) and the National Health and Medical Research Council (Australia) as part of the International Collaborative Research Grants Scheme awarded jointly to NIHRD and MSHR. The arginine studies were a central component of the combined NIHRD-MSHR submission to the Wellcome Trust and NHMRC, and were subject to international peer review. *No funding has or will be received from any companies manufacturing L-arginine.* L-arginine is prepared as an inexpensive generic commercially available drug (see generic product information attached).

## Authorship/ownership of the study: will be shared as per International Committee of Medical Journal Editors 2003 criteria.

*Certificate of analysis for intraveneous L-arginine:* This was provided to Indonesian FDA and approved prior to stage 2. We will be using exactly the same preparation of L-arginine (Pharmalab, NSW, Australia) for stage 3: the COA for the batch to be used in Stage 3 is attached to this protocol.

**References**

1. Guerin PJ, Olliaro P, Nosten F, et al. Malaria: current status of control, diagnosis, treatment, and a proposed agenda for research and development. *Lancet Infect Dis* 2002;**2**(9)**:**564-73.

2. Golenser J, McQuillan J, Hee L, Mitchell AJ, Hunt NH. Conventional and experimental treatment of cerebral malaria. *Int J Parasitol* 2006;**36**(5)**:**583-93.

3. Dondorp A, Nosten F, Stepniewska K, Day N, White N. Artesunate versus quinine for treatment of severe falciparum malaria: a randomised trial. *Lancet* 2005;**366**(9487)**:**717-25.

4. Moncada S, Higgs A. The L-arginine-nitric oxide pathway. *New England Journal of Medicine* 1993;**329:**2002-2012.

5. Weinberg J. Nitric oxide production and nitric oxide synthase type 2 expression by human mononuclear phagocytes: a review. *Molec Med* 1998;**4:**557-591.

6. Degroote MA, Fang FC. NO inhibitions. Antimicrobial properties of nitric oxide. *Clinical Infectious Diseases* 1995;**21**(Suppl 2)**:**S 162-S 165.

7. Anstey N, Weinberg J, Granger D. Nitric oxide and malaria. In: Fang F, ed. Nitric oxide and infection. New York: Plenum Publishing Corp, 1999: 311-341.

8. Wu G, Morris S. Arginine metabolism: nitric oxide and beyond. *Biochem J* 1998;**336:**1-17.

9. Xia Y, Roman LJ, Masters BS, Zweier JL. Inducible nitric-oxide synthase generates superoxide from the reductase domain. *J Biol Chem* 1998;**273**(35)**:**22635-9.

10. Vasquez-Vivar J, Kalyanaraman B, Martasek P, et al. Superoxide generation by endothelial nitric oxide synthase: the influence of cofactors. *Proc Natl Acad Sci U S A* 1998;**95**(16)**:**9220-5.

11. Wang W, Wang S, Yan L, et al. Superoxide production and reactive oxygen species signaling by endothelial nitric-oxide synthase. *J Biol Chem* 2000;**275**(22)**:**16899-903.

12. Morris CR, Kuypers FA, Larkin S, Vichinsky EP, Styles LA. Patterns of arginine and nitric oxide in patients with sickle cell disease with vaso-occlusive crisis and acute chest syndrome. *J Pediatr Hematol Oncol* 2000;**22**(6)**:**515-20.

13. Morris CR, Kuypers FA, Larkin S, et al. Arginine therapy: a novel strategy to induce nitric oxide production in sickle cell disease. *Br J Haematol* 2000;**111**(2)**:**498-500.

14. Yu YM, Ryan CM, Castillo L, et al. Arginine and ornithine kinetics in severely burned patients: increased rate of arginine disposal. *Am J Physiol Endocrinol Metab* 2001;**280**(3)**:**E509-17.

15. Freund H, Atamian S, Holroyde J, Fischer JE. Plasma amino acids as predictors of the severity and outcome of sepsis. *Ann Surg* 1979;**190**(5)**:**571-6.

16. Argaman Z, Young V, Noviski N, et al. Arginine and nitric oxide metabolism in critically ill septic pediatric patients. *Crit Care Med* 2003;**31**(2)**:**February: in press.

17. Boger RH, Bode-Boger SM. The clinical pharmacology of L-arginine. *Annu Rev Pharmacol Toxicol* 2001;**41:**79-99.

18. Bode-Boger SM, Boger RH, Galland A, Tsikas D, Frolich JC. L-arginine-induced vasodilation in healthy humans: pharmacokinetic-pharmacodynamic relationship. *Br J Clin Pharmacol* 1998;**46**(5)**:**489-97.

19. Anstey NM, Weinberg JB, Hassanali MY, et al. Nitric oxide in Tanzanian children with malaria: inverse relationship between malaria severity and nitric oxide production/nitric oxide synthase type 2 expression. *J Exp Med* 1996;**184**(2)**:**557-67.

20. Perkins DJ, Kremsner PG, Schmid D, Misukonis MA, Kelly MA, Weinberg JB. Blood mononuclear cell nitric oxide production and plasma cytokine levels in healthy gabonese children with prior mild or severe malaria. *Infect Immun* 1999;**67**(9)**:**4977-81.

21. Hobbs MR, Udhayakumar V, Levesque MC, et al. A new NOS2 promoter polymorphism associated with increased nitric oxide production and protection from severe malaria in Tanzanian and Kenyan children. *Lancet* 2002;**360**(9344)**:**1468-75.

22. Anstey NM, Boutlis CS, Tjitra E, et al. Inverse association between disease severity and systemic nitric oxide (NO) production and mononuclear cell inducible NO synthetase (NOS2) expression in Indonesian adults with malaria suggests anti-disease effects of NO. *Am J Trop Med Hyg* 2001;**65**(3)**:**Suppl.: 400.

23. Florquin S, Amraoui Z, Dubois C, Decuyper J, Goldman M. The protective role of endogenously synthesized nitric oxide in staphylococcal enterotoxin B-induced shock in mice. *J Exp Med* 1994;**180:**1153-1158.

24. De Caterina R, Libby P, Peng HB, et al. Nitric oxide decreases cytokine-induced endothelial activation. Nitric oxide selectively reduces endothelial expression of adhesion molecules and proinflammatory cytokines. *J Clin Invest* 1995;**96**(1)**:**60-8.

25. Maneerat Y, Viriyavejakul P, Punpoowong B, et al. Inducible nitric oxide synthase expression is increased in the brain in fatal cerebral malaria [In Process Citation]. *Histopathology* 2000;**37**(3)**:**269-77.

26. Dupont WD, Plummer WD, Jr. Power and sample size calculations. A review and computer program. *Control Clin Trials* 1990;**11**(2)**:**116-28.

27. Pareznik R, Knezevic R, Voga G, Podbregar M. Changes in muscle tissue oxygenation during stagnant ischemia in septic patients. *Intensive Care Med* 2006;**32**(1)**:**87-92.

28. Tran TH, Day NP, Nguyen HP, et al. A controlled trial of artemether or quinine in Vietnamese adults with severe falciparum malaria. *N Engl J Med* 1996;**335**(2)**:**76-83.

29. Beal SL, Sheiner L. NONMEM User's Guide, parts I-VII. 1992**:**San Francisco: NONMEM Project Group.

30. Lopansri B, Anstey N, Weinberg J, et al. Low plasma arginine concentrations in children with cerebral malaria and decreased nitric oxide production. *Lancet* 2003;**361:**February: in press.

31. Hyland K, Howells DW. Analysis and clinical significance of pterins. *J Chromatogr* 1988;**429:**95-121.

32. Recommendations for standardized procedures for the on-line and off-line measurement of exhaled lower respiratory nitric oxide and nasal nitric oxide in adults and children-1999. This official statement of the American Thoracic Society was adopted by the ATS Board of Directors, July 1999. *Am J Respir Crit Care Med* 1999;**160**(6)**:**2104-17.

33. Spronk PE, Ince C, Gardien MJ, Mathura KR, Oudemans-van Straaten HM, Zandstra DF. Nitroglycerin in septic shock after intravascular volume resuscitation. *Lancet* 2002;**360**(9343)**:**1395-6.

34. Girardis M, Rinaldi L, Busani S, Flore I, Mauro S, Pasetto A. Muscle perfusion and oxygen consumption by near-infrared spectroscopy in septic-shock and non-septic-shock patients. *Intensive Care Med* 2003;**29**(7)**:**1173-6.

35. De Blasi RA, Palmisani S, Alampi D, et al. Microvascular dysfunction and skeletal muscle oxygenation assessed by phase-modulation near-infrared spectroscopy in patients with septic shock. *Intensive Care Med* 2005;**31**(12)**:**1661-8.

36. Galban C, Montejo JC, Mesejo A, et al. An immune-enhancing enteral diet reduces mortality rate and episodes of bacteremia in septic intensive care unit patients. *Crit Care Med* 2000;**28**(3)**:**643-8.

37. Annane D, Sanquer S, Sebille V, et al. Compartmentalised inducible nitric-oxide synthase activity in septic shock. *Lancet* 2000;**355**(9210)**:**1143-8.

38. Massara F, Martelli S, Cagliero E, Camanni F, Molinatti GM. The hypophosphatemic and hyperkalemic effect of arginine in man. *J Endocrinol Invest* 1980;**3**(2)**:**177-80.

39. Baker GL, Franklin JD. Management of arginine monohydrochloride extravasation in the forearm. *South Med J* 1991;**84**(3)**:**381-4.

40. Tiwary CM, Rosenbloom AL, Julius RL. Anaphylactic reaction to arginine infusion. *N Engl J Med* 1973;**288**(4)**:**218.

41. Palmer JP, Walter RM, Ensinck JW. Arginine-stimulated acute phase of insulin and glucagon secretion. I. in normal man. *Diabetes* 1975;**24**(8)**:**735-40.

42. Barbul A. Arginine: biochemistry, physiology, and therapeutic implications. *JPEN J Parenter Enteral Nutr* 1986;**10**(2)**:**227-38.

43. Hertz P, Richardson JA. Arginine-induced hyperkalemia in renal failure patients. *Arch Intern Med* 1972;**130**(5)**:**778-80.

44. Adrogue HJ, Madias NE. Management of life-threatening acid-base disorders. Second of two parts. *N Engl J Med* 1998;**338**(2)**:**107-11.

45. Schlaich MP, Jacobi J, John S, Delles C, Fleischmann I, Schmieder RE. Is l-arginine infusion an adequate tool to assess endothelium-dependent vasodilation of the human renal vasculature? *Clin Sci (Lond)* 2000;**99**(4)**:**293-302.

46. Koller-Strametz J, Wolzt M, Fuchs C, et al. Renal hemodynamic effects of L-arginine and sodium nitroprusside in heart transplant recipients. *Kidney Int* 1999;**55**(5)**:**1871-7.

47. WHO. Severe falciparum malaria. *Trans R Soc Trop Med Hyg* 2000;**94, suppl 1:**S1-S90.

| **TABLE 1: IV L-arginine or saline over 8 hr** | D0 Clinic sample  mins | 0 | 5-10  mins | 60 | 120 | 240 | 480 | 540 | 720 | Day 1 | Day 1 | Day 2 | Day 2  § | Day 3  § | Day 4  § | Day ≥5  § |
| --- | --- | --- | --- | --- | --- | --- | --- | --- | --- | --- | --- | --- | --- | --- | --- | --- |
| Time (hrs) | -5 to -1 | 0 | 0.1 | 1 | 2 | 4 | 8 | 9 | 12 | 16  (14-20) | 24  (21-28) | 32 | 40-48 | ±72 | 96 | 120 |
| Blood volume |  | 6 | 1 | 1 | 2 | 2 | 6 | 2 | 2 | 0.2 | 2 | 0.2 | 2 | 2 | †Clinical blood | †Clinical blood |
| Vital signs (BP, PR, RR): q15 min until 120 min, then q20-30 min until 9 hrs; then hrly for 2hrs, then 2-4 hrly |  | X |  | X | X | X | X | X | X | X | X | X | X | X | X | X |
| Clinical symptoms/signs  As per vital signs |  | X |  | X | X | X | X | X | X | X | X | X | X | X | X | X |
| iSTAT EC8+ #* or electrolytes in plasma** | X | X |  | X | X | X | X | X | X | X | X | X | X | X | †X | †X |
| Phosphate, Ca++ ** |  | X |  |  | X | X | X | X | X |  |  |  |  |  |  |  |
| Thick/thin film* | X | X |  |  |  |  | X | X | X | X | X |  | X | X | †X | †X |
| Lactate (CG4+)* ∞ |  | X |  |  |  |  | X |  | X | X | X | X § | X | X | †X | †X |
| LFTs, CK, Creatinine** |  | X |  |  |  |  |  |  |  | X |  | X |  | X | †x | †x |
| Exhaled NO* |  | X |  | X | X |  | X | X | X | X | X | X |  | X | X | X |
| DLCO* |  |  |  |  |  |  |  |  |  |  |  |  |  | X |  |  |
| NIRS (St02) * |  | X |  | X |  |  | X |  | X |  | X |  | X | X | X | X |
| OPS* |  | X |  | X |  |  | X |  |  |  | X |  | X | X | X |  |
| Endothelial function* |  | X |  | X |  |  | X |  | X |  | X |  | X | X | X | X |
| Amino acids (incl. arginine), NOx, haptoglobin, oxidant markers, cytokines** |  | X | X | X | X | X | X |  |  |  | X | X | X | X | X | X |
| T cell function and phenotype |  | X |  |  |  |  | X |  |  |  |  |  |  |  |  |  |
| Urine (pterins, NOx)** |  | X |  |  |  |  | X*** |  |  |  | X*** |  | X*** | X*** |  |  |

**Footnotes for table 1:**

Grey shading: duration of L-arginine infusion

# iSTAT EC8+ is a bedside biochemistry cartridge which uses only several microlitres to measure Na^+^, K^+^, Cl^-^, pH, PCO_2_, urea nitrogen (BUN)/urea, glucose, hematocrit, TCO_2_, bicarbonate, Base excess, anion gap, hemoglobin. Baseline measure of methaemoglobin at RSMM laboratory to compare with co-oximetry.

ECG and co-oximetry will be monitored continuously during the first 2 hours of the L-arginine infusion (at which time steady state levels of L-arginine are achieved). Temperature will also be monitored 4-6 hourly until 20 hours.

‡Clinical symptom and sign assessment: questioned on presence of headache, lightheadedness, nausea, vomiting, flushing, numbness, difficulty breathing, pain at infusion site; and examined for rash.

NIRS: near infrared spectroscopy: microvascular function test

OPS: Orthogonal polarizing spectroscopy (rectal probe): quantitation of microvascular obstruction

DLCO: gas transfer as performed in stage 2 (to correlate with exhaled NO on day 3: will test whether pulmonary NO production is associated with improved gas transfer)

**Location of each testing procedure:** A strength of this study is that all the endothelial physiology measurements, all the tissue oxygenation saturation and microvascular obstruction measurements, all the parasite counts, the lactate and phosphate measurements, and most of the other biochemical measurements (eg iSTAT bedside biochemistry) will be undertaken **on site** at RSMM in Timika (either at the bedside or at the RSMM lab), with on-the-job training of all local and visiting staff involved. The asterisks (* or **) identify which measurements will be done at RSMM in Timika (*) and which at MSHR (**) or University of Sydney/NSW (oxidant stress measurements, microparticles).

∞ will be measured until normalised

† will be measured if clinically indicated

*performed at RSMM.

** performed in Darwin

*** Urine will be collected at approx 4 hours, and at each time voided thereafter.

§ if patient still in hospital

Clinical blood: blood requested by treating doctor for clinical purposes. If a severe malaria patient remains in hospital on day 4 and 5, it is usual for the RSMM doctor to request renal function, glucose and electrolytes. If blood is requested by RSMM doctors, we will use the RSMM hospital results and store residual plasma for amino acid levels from remaining plasma. RSMM doctors routinely use the iSTAT data we collect for research purposes (electrolytes, glucose, renal function and lactate) for their clinical management.

**Appendix 1**

### List of Abbreviations

ATS: American Thoracic Society

CAT 1: Cationic amino acid transporter 1

CAT 2: Cationic amino acid transporter 2

CK: Creatine Kinase

CL: Clearance

Cl^-^: Chloride

CM: Cerebral Malaria

DLCO: Non-invasive Gas transfer assessment (diffusion of CO)

FMD: Flow mediated dilatation

GMP: Good Manufacturing Practice

GSH: Plasma glutathione

GSSG: Oxidised Glutathione

HCO3-: Bicarbonate

iSTAT: Brand of Portable Clinical Analyzer

K+: Potassium

Km: Equilibrium constant

LFTs: Liver Function Tests

MSHR: Menzies School of Health Research

NIHRD: National Institute for Health Research and Development

NONMEM: Computer software program to model non-linear pharmacokinetics of drugs.

NIRS: Near infrared spectroscopy

NO: Nitric oxide

NOS1: Nitric oxide Synthase 1 or Neuronal Nitric oxide Synthase

NOS2: Nitric oxide Synthase 2 or Inducible Nitric oxide Synthase

NOS3: Nitric oxide Synthase 3 or Endothelial Nitric oxide Synthase

NOx: Nitric oxide metabolites, nitrate plus nitrite

OPS: Orthogonal Polarising Spectroscopy

PAT: Peripheral Arterial Tonometry

PBMCs: Peripheral blood mononuclear cells

PK: Pharmacokinetic (change in L-arginine concentration over time)

PD: Pharmacodynamic (change in L-arginine effect over time)

RSMM: Rumah Sakit Mitra Masyarakat

sICAM-1: soluble intercellular adhesion molecule 1

SEAQUAMAT: South East Asian multi-center study of QUinine versus Artesunate for severe MAlaria Trial.

SM: Severe *Plasmodium falciparum* Malaria

StO2: Tissue Oxygen Saturation

SCD: Sickle Cell Disease

VOCS: Vaso-occlusive syndrome (found in SCD)

UF: Uncomplicated *Plasmodium falciparum* malaria

WHO: World Health Organization

**Appendix 2**

**Study groups for the further descriptive studies of the microvascular function tests being used in the RCT above:**

**Adult Patients:**

*Severe Malaria (SM)*

These will be the patients enrolled in the RCT

*Moderately severe falciparum malaria (MSM)*:

Inclusion criteria:

1. age 18-60 years
2. *P. falciparum* parasitemia (1,000-100,000 parasites/ul).
3. Clinical syndrome consistent will malaria associated with documented fever (axillary temperature ≥ 38^o^C) or self-reported history of fever in the last 48 hours with no other cause present
4. Commencement of parenteral antimalarial therapy < 18 hours previously
5. An indication for hospital admission [eg relative cannot stay at home to look after them or supervise their treatment at home, inability to tolerate oral therapy eg. vomiting], but not having the warning signs or severe malaria criteria in “exclusion criteria” below
6. Informed consent obtained

Exclusion criteria:

1. pregnancy or lactation
2. warning signs of altered mental state and inability to sit unaided
3. features of severe/complicated malaria^47^ (see “severe malaria” above)
4. diabetes
5. systolic BP <80 mmHg
6. serious pre-existing diseases (cardiac, hepatic, kidney and lung)
7. clinical evidence of concurrent bacterial infection
8. medications as per severe malaria exclusion criteria

*Adult Healthy Controls* *(HC)*

1. age 18-60 years
2. no *Plasmodium* parasitemia
3. no clinical symptoms consistent with malaria or other infections/acute inflammation
4. informed consent obtained
5. no pregnancy or lactation
6. no diabetes
7. systolic BP ≥ 80 mmHg
8. no serious underlying disease (cardiac, hepatic, kidney)
9. not immediately genetically related to a patient included in the study (eg parent, sibling or cousin)

*Adult Sepsis (SIRS)*:

Inclusion criteria:

1. age 18-60 years
2. informed consent
3. suspected or proven infection, AND three or more of the following SIRS criteria:
4. hody temperature >38^o^C or <36^o^C
5. heart rate >90 beats/minute
6. respiratory rate >20 breaths/min and/or P_a_CO2 <32mmHg
7. white blood cell count >12,000 cells/ml or <4,000 cells/ml or >10% band forms

Exclusion criteria:

- 1. pregnancy or lactation
  2. infection with any plasmodium species
  3. diabetes
  4. serious pre-existing diseases (cardiac, hepatic, kidney and lung)

**Children:**

*Cerebral Malaria (CM)*, defined by modified WHO criteria^28^:

Inclusion criteria:

1. age 4-12 years
2. informed consent obtained
3. time of commencement of artesunate ≤18 hrs
4. any level of *P. falciparum* parasitemia
5. cerebral malaria (Blantyre coma score ≤2)
6. no evidence of concurrent bacterial infection
7. Hb ≥5 g/dL

*Uncomplicated falciparum malaria (UF)*:

Inclusion criteria:

1. age 4-12 years
2. informed consent obtained
3. time of commencement of hospital antimalarial ≤18 hrs
4. *P. falciparum* parasitemia (1,000-100,000 parasites/ul).
5. Clinical syndrome consistent will malaria associated with documented fever (axillary temperature ≥ 38^o^C) or self-reported history of fever in the last 48 hours with no other cause present
6. no evidence of WHO criteria for severe malaria
7. No warning signs of prostration or aletered mental state
8. no evidence of concurrent bacterial infection
9. Hb ≥7 g/dL

*Healthy Controls* *(HC)*

Inclusion criteria:

1. age 4-12 years
2. informed consent obtained
3. no Plasmodium infection
4. no fever or history of fever in past 7 days
5. no evidence of concurrent bacterial infection
6. Hb ≥7 g/dL

Information Sheet:

“L-arginine in severe malaria: phase 2A/2B”

Malaria is a common infection in Indonesia and may cause serious illness. This form tells you about a research project that is looking at whether a natural substance produced by the body called L-arginine can be given as an extra treatment to help people get better from severe malaria.

In this study you or your relative will receive the standard treatment recommended by the Ministry of Health of the Republic of Indonesia. In addition we will give you or your relative fluid through a vein. This fluid will either contain L-arginine or just fluid alone.

If you or your relatives agree to join this research study we will use a small needle to take a small amount of blood from a vein in yours or your relative’s arm. This is momentarily painful with a small risk of bleeding or bruising. Some of this blood will be used to treat and monitor the infection, and some of the blood will be used for malaria research. It will be examined for L-arginine, nitric oxide and substances related to the malaria infection. We will leave this cannula in the vein overnight while you or your relative are being treated for malaria, and used this to take smaller amounts of your blood up to 12 more times over 3 days. These blood samples will be used by the doctor to treat and monitor the infection and some will be used for malaria research. The total amount of blood taken will be no more than 29mls (or 6 teaspoons) which is a safe amount.

We will also measure the pulse, blood pressure and heart tracing and ask you or your relative questions every few minute when we give the L-arginine and then less frequently after that. We will ask to obtain a small amount of urine now and whenever you or your relative need to pass urine. We will also ask you or your relative to breath into a tube every 30 minutes at first for 1-2 hours and later every few hours to check the amount of nitric oxide in the breath and how well the lungs work. These are tests used routinely in hospitals to check people’s lungs and do not hurt.

We will also test the function of the blood vessels at the finger and thumb. This technique is safe and painless and does not cause any harm. The test of your finger/thumb will be done resting, lying down and relaxing. We will then put a blood pressure cuff on the forearm and inflate the cuff for 5 minutes. When the cuff is let down, we will continue testing for another 5 minutes. Inflating the cuff will be uncomfortable, like having a blood pressure recorded but will cause no damage. We will repeat these tests one and eight hours later and again each day whiles you or your relative are in hospital.

We will look at the lining of the back passage (rectum) to see if the blood vessels are blocked up. We will ask you to roll on the side. We will insert a probe in the back passage like a thermometer to look at blood vessels inside. This will last 1-2 minutes. We will repeat this several times over the next 3 days to see how these blood vessels recover.

You or your relative’s participation in the research will not cost you or your family any money. Your doctor may recommend some other tests and procedures to better diagnose and treat your infection. If you or your relatives decide not to participate in this study, you will receive the standard treatment and care for malaria. You or your relative may wish to withdraw from the study at any time and still receive standard treatment and care. There will no name or other identification in any study report which will be published later on. You may contact Dr Enny on 901-301-882 at any time to answer any questions about the study. If you have any concerns about your rights or the conduct of the study you may contact Dr Liliana Kurniawan on 21-425 9860 or Maria Scarlett on 61-8-8922-7922.

**Benefits of study:** Most people with severe malaria will get better with drugs to kill the malaria parasites. Giving the extra treatment with L-arginine may help more people get better but we don’t know.

**Risks from the study:** L-arginine is a natural substance in the body and in many foods, but is also available as an injection in hospitals. It has been used a standard drug in hospitals for many years in larger doses than we will use today. Even when used in bigger doses than we will use it is usually very safe. The amount of L-arginine we will give has previously been shown to be safe in people with milder malaria. Sometimes large doses can cause the blood pressure to go down and sometimes people may feel dizzy, feel up or down but it is unusual for this to cause problems. It can also cause irritation of the veins or very rarely an allergic reaction. Because we will use smaller doses we do not expect these to be a problem for you. We will watch for any side-effects and can give you treatment if any of them develop.

“L-arginine in severe malaria:study 2A/2B”

This form means I can say “No”

I have read and understood the information sheet attached, and have been given the opportunity to discuss it and ask questions. I agree that my samples can be used in the research outlined on the information sheet. I understand that I do not have to participate in this study. If I do not participate in this study I will receive the usual treatment.

I also DO/DO NOT agree for my samples to be stored indefinitely for potential future use for research related to how people respond to or are protected from malaria. I understand that my stored samples will not be used for any other purpose.

I also DO/DO Not agree to have a examination of the back passage/rectum

By my signature below I consent to participate:

______________________________ _______________________________

Name of study subject / Date Signature of study subject

_______________________________ ________________________________

Name of person obtaining consent/ Signature of person obtaining consent

Date

“L-arginine in severe malaria:study 2A/2B”

Consent form for next of kin

This form means I can say “No” for my relative

I have read and understood the information sheet attached, and have been given the opportunity to discuss it and ask questions. I agree that my relative’s samples can be used in the research outlined on the information sheet. I understand that I do not have to consent for my relative to participate in this study. If I do not wish them participate in this study they will receive the usual treatment.

I also DO/DO NOT agree for their samples to be stored indefinitely for potential future use for research related to how people respond to or are protected from malaria. I understand that their stored samples will not be used for any other purpose.

I also DO/DO Not agree for my relative to have a examination of the back passage/rectum

By my signature below I give consent for my relative to participate:

______________________________

Name of study subject / Date

______________________________ ________________________________

Name of next of kin/ Date Signature of next of kin

_______________________________ ________________________________

Name of person obtaining consent/ Signature of person obtaining consent

Date

­­ Information Sheet:

“L-arginine and blood vessel function

in healthy controls/malaria/sepsis”

Malaria is a common infection in Indonesia and may cause serious illness. This form tells you about a research project that is looking at whether a natural substances produced by the body called L-arginine and nitric oxide affect the function of blood vessels in healthy people against those with malaria or other infections and what happens to them as people recover from malaria.

This study will see how quickly levels of L-arginine come back to normal as you or your relative get better from your illness and whether this is related to the amount of nitric oxide made. We need to know this so that we can plan how much L-arginine we may need to give people in the future if they get severe malaria.

If you agree or you agree on behalf of your relative for them to join this research study we will use a small needle to take a small amount of blood from a vein in the arm. This is momentarily painful with a small risk of bleeding or bruising. Some of this blood will be used to treat and monitor the infection, and some of the blood will be used for malaria research. It will be examined for L-arginine, nitric oxide and substances related to the malaria infection. We will also take smaller amounts of blood up to two more times over 2 days. The total amount of blood taken will be no more than 14mls (or 3 teaspoons) which is a safe amount.

We will ask to obtain a small amount of urine now and whenever urine is excreted. We will also ask you or your relative to breathe into a tube every few hours to check the amount of nitric oxide in the breath and how well the lungs work. These are tests used routinely in hospitals to check people’s lungs and do not hurt.

We will also test the function of the blood vessels at the finger and thumb. This technique is safe and painless and does not cause any harm. The test of the finger/thumb will be done with resting, lying down and relaxing. We will then put a blood pressure cuff on the forearm and inflate the cuff for 5 minutes. When the cuff is let down, we will continue testing for another 5 minutes. Inflating the cuff will be uncomfortable, like having the blood pressure recorded but will cause no damage. We will repeat these tests one and eight hours later and again each day while you or your relative are in hospital.

This is research to try and understand how people recover from malaria and infections. You or your relative will receive the standard treatment recommended by the Ministry of Health of the Republic of Indonesia. Participation in the research will not cost you or your relative’s family any money. Your doctor may recommend some other tests and procedures to better diagnose and treat your infection. If you decide not to participate in this study or decide not to have your relative participate, you will receive the standard treatment and care for malaria. You or your relative may wish to withdraw from the study at any time and still receive standard treatment and care. There will no name or other identification in any study report which will be published later on. You may contact Dr Enny on 901-301-882 at any time to answer any questions about the study. If you have any concerns about your rights or the conduct of the study you may contact Dr Liliana Kurniawan on 21-425 9860 or Maria Scarlett on 61-8-8922-7922.

**Benefits of study:** If you or your relative have malaria you will receive the usual treatment for malaria or infections and will not otherwise receive direct benefit from the study. However by understanding how blood vessels function during recovery from malaria and other infections and it relationship to blood levels of L-arginine, we hope to know how much L-arginine to give people with malaria in the future.

CONSENT TO PARTICIPATE IN A MALARIA RESEARCH STUDY

“L-arginine and blood vessel function in healthy controls/malaria/sepsis”

This form means I can say “No”

I have read and understood the information sheet attached, and have been given the opportunity to discuss it and ask questions. I agree that my samples can be used in the research outlined on the information sheet. I understand that I do not have to participate in this study. If I do not participate in this study I will receive the usual treatment.

I also DO/DO NOT agree for my samples to be stored indefinitely for potential future use for research related to how people respond to or are protected from malaria. I understand that my stored samples will not be used for any other purpose.

By my signature below I consent to participate:

______________________________ _______________________________

Name of study subject / Date Signature of study subject/ next of kin

_______________________________ ________________________________

Name of person obtaining consent/ Signature of person obtaining consent

Date

“L-arginine and blood vessel function in healthy controls/malaria/sepsis”

Consent form for next of kin

This form means I can say “No” for my relative

I have read and understood the information sheet attached, and have been given the opportunity to discuss it and ask questions. I agree that my relative’s samples can be used in the research outlined on the information sheet. I understand that I do not have to consent for my relative to participate in this study. If I do not wish them participate in this study they will receive the usual treatment.

I also DO/DO NOT agree for their samples to be stored indefinitely for potential future use for research related to how people respond to or are protected from malaria. I understand that their stored samples will not be used for any other purpose.

I also DO/DO Not agree for my relative to have a examination of the back passage/rectum

By my signature below I give consent for my relative to participate:

______________________________

Name of study subject / Date

______________________________ ________________________________

Name of next of kin/ Date Signature of next of kin

_______________________________ ________________________________

Name of person obtaining consent/ Signature of person obtaining consent

Date

Information Sheet:

“L-arginine and blood vessel function

in children with malaria”

Malaria is a common infection in Indonesia and may cause serious illness. This form tells you about a research project that is looking at whether a natural substances produced by the body called L-arginine and nitric oxide affect the function of blood vessels in healthy children against those with malaria or other infections and what happens to them as children recover from malaria.

This study will see how quickly levels of L-arginine come back to normal as children get better from your illness and whether this is related to the amount of nitric oxide they make. We need to know this so that we can plan how much L-arginine we may need to give children in the future if they get severe malaria.

If you agree for your child to join this research study we will use a small needle to take a small amount of blood from a vein in your arm. This is momentarily painful. We will use this to take a small amount of blood (7ml or 1½ teaspoons). Some of this blood will be used to treat and monitor your infection, and some of the blood will be used for malaria research. It will be examined for L-arginine, nitric oxide and substances related to his/her malaria infection. We will also take smaller amounts of blood up to two more times over 2 days. The total amount of blood taken will be no more than 11mls (or 2 teaspoons) which is a safe amount.

We will ask to obtain a small amount of your child’s urine now and each day.

We will also test the function of the blood vessels at the finger and thumb. This technique is safe and painless and does not cause any harm. The test of your finger/thumb will be done with your child resting, lying down and relaxing. We will then put a blood pressure cuff on his/her forearm and inflate the cuff for 5 minutes. When the cuff is let down, we will continue testing for another 5 minutes. Inflating the cuff will be uncomfortable, like having your blood pressure recorded but will cause no damage. Then there will be a break for 10-15 minutes. We will then do another test with him/her relaxing. We will repeat these tests each day while you are in hospital.

This is research to try and understand how children recover from malaria and infections. You will receive the standard treatment recommended by the Ministry of Health of the Republic of Indonesia. Your child’s participation in the research will not cost you or your family any money. Your doctor may recommend some other tests and procedures to better diagnose and treat your infection. If you decide not to participate in this study, your child will receive the standard treatment and care for malaria. You withdraw your child from the study at any time and still receive standard treatment and care.

There will no name or other identification in any study report which will be published later on. You may contact Dr Enny on 901-301-882 at any time to answer any questions about the study. If you have any concerns about your rights or the conduct of the study you may contact Dr Liliana Kurniawan on 21-425 9860 or Maria Scarlett on 61-8-8922-7922.

**Benefits of study:** If your child has malaria he/she will receive the usual treatment for malaria or infections and will not otherwise receive direct benefit from the study. However by understanding how blood vessels function during recovery from malaria and other infections and it relationship to blood levels of L-arginine, we hope to know how much L-arginine to give children with malaria in the future.

CONSENT TO PARTICIPATE IN A MALARIA RESEARCH STUDY

“L-arginine and blood vessel function in children with malaria”

This form means I can say “No”

I have read and understood the information sheet attached, and have been given the opportunity to discuss it and ask questions. I agree that my child’s samples can be used in the research outlined on the information sheet. I understand that my child does not have to participate in this study. If my child does not participate in this study he will receive the usual treatment.

I also DO/DO NOT agree for my child’s samples to be stored indefinitely for potential future use for research related to how people respond to or are protected from malaria. I understand that my child’s stored samples will not be used for any other purpose.

By my signature below I consent for my child to participate:

______________________________ _______________________________

Name of study subject / Date Signature of parent/ guardian

_______________________________ ________________________________

Name of person obtaining consent/ Signature of person obtaining consent

Date
